# Brain idiosyncrasy during biological-motion perception is amplified in autistic individuals with intellectual impairment

**DOI:** 10.1186/s11689-026-09683-3

**Published:** 2026-03-25

**Authors:** Michael Cheng, Colin Hawco, Daniel Yang, Hsing-Chang Ni, Chun-Hung Yeh, Jung-Chi Chang, En-Nien Tu, Mei-Yun Hsu, Yu-Yu Wu, Tai-Li Chou, Susan Shur-Fen Gau, Hsiang-Yuan Lin

**Affiliations:** 1 Institute of Medical Science, Temerty Faculty of Medicine, University of Toronto, Toronto, ON Canada; 2 Azrieli Adult Neurodevelopmental Centre, Campbell Family Mental Health Research Institute, Centre for Addiction and Mental Health, 1025 Queen St West – 3314, Toronto, ON M6J 1H4 Canada; 3 Campbell Family Mental Health Research Institute, Centre for Addiction and Mental Health, Toronto, ON Canada; 4 Department of Psychiatry, Temerty Faculty of Medicine, University of Toronto, Toronto, ON Canada; 5 Independent Scholar (with George Washington University, USA While This Study Was Being Conducted), Washington D.C., USA; 6 Department of Psychiatry, Chang, Gung Memorial Hospital at Linkou, Taoyuan, Taiwan; 7 College of Medicine, Chang Gung University, Taoyuan, Taiwan; 8 Department of Medical Imaging and Radiological Sciences, Chang Gung University, Taoyuan, Taiwan; 9 Department of Psychiatry, National Taiwan University Hospital and College of Medicine, Taipei, Taiwan; 10 Department of Psychiatry, University of Oxford, Oxford, UK; 11 Department of Psychiatry, Keelung Chang Gung Memorial Hospital, Keelung, Taiwan; 12 YuNing Clinic, Taipei, Taiwan; 13 Department of Psychology, National Taiwan University, Taipei, Taiwan

**Keywords:** Autism spectrum condition, Intellectual impairment, Brain idiosyncrasy, Biological motion, FMRI, Heterogeneity, Social cognition

## Abstract

**Background:**

Neuroimaging research in autism spectrum condition (ASC) often overlooks brain idiosyncrasy by focusing on group averages and frequently excludes individuals with co-occurring intellectual impairment (II).

**Methods:**

We investigated functional MRI correlates of passive biological motion (BM) perception, comparing typically developing controls (TDC; *n* = 33), autistic individuals without II (intellectually able; ASC-IA; *n* = 28), and autistic individuals with II (ASC-II; *n* = 19; defined by IQ or adaptive function < 85).

**Results:**

While standard group-average analyses revealed the expected BM-sensitive regions (e.g., bilateral posterior superior temporal sulci, cuneus) in the TDC and ASC-IA groups, the ASC-II group showed no consistent group-level activation pattern and exhibited greater activation in the right intraparietal sulcus compared to the ASC-IA group. Using a correlational distance-based metric, we quantified brain idiosyncrasy (“whole-sample brain variability”, Variability_Whole_), representing the deviance of an individual's activation pattern from others. Brain activity in the ASC-II group was significantly more idiosyncratic than the ASC-IA and TDC groups. Furthermore, Variability_Whole_ showed significant transdiagnostic correlations with multiple cognitive and behavioural domains relevant to autism, including social difficulties, repetitive behaviours, non-verbal IQ, executive function, sensory hyper/hyposensitivity, ADHD symptoms, and adaptive function.

**Conclusions:**

Key limitations include the cross-sectional design and the use of a passive viewing task without a concurrent behavioral measure to directly link brain findings to task performance. These findings highlight substantial brain heterogeneity within the autism spectrum, particularly in the understudied ASC-II subgroup, and suggest that individual differences in brain processing patterns, rather than solely group-average differences, are critically linked to clinical and cognitive phenotypes.

**Supplementary Information:**

The online version contains supplementary material available at 10.1186/s11689-026-09683-3.

## Background

Autism spectrum condition (ASC) is characterized by profound heterogeneity spanning genetics, etiology, and the resulting cognitive and behavioural phenotypes [57]. This heterogeneity challenges conventional group-average neuroimaging approaches [23, 56] and has spurred interest in brain idiosyncrasy – individual-specific variations in brain structure and function [40, 66] – as a critical dimension for direct investigation [55].

Traditional neuroimaging paradigms in ASC research have often prioritized group-average effects [23, 56], inadvertently marginalizing the rich information embedded within brain idiosyncrasy [6, 10, 12, 20, 40, 42, 66, 69, 93]. This focus is compounded by a historical research bias towards recruiting intellectually able autistic individuals (ASC-IA) and excluding those with co-occurring intellectual impairment (ASC-II) [48], due to practical challenges concerning consent, task compliance, and head motion artefacts, alongside potential confounds of co-occurring conditions. However, given that approximately 30–40% of autistic individuals have co-occurring II [88], this systemic underrepresentation severely limits the ecological validity and generalizability of findings [48].

A relevant domain for probing social brain function in ASC is biological motion (BM) perception: the ability to visually perceive and interpret human movement and infer socially relevant information. BM is often studied using point-light displays [52]. These minimalist stimuli convey rich social information (e.g., actions, intentions, emotions) purely through movement patterns [39, 43, 49]. An intriguing puzzle is the interaction between BM perception and general intelligence. In neurotypical individuals, BM perception appears to engage a relatively specialized network (posterior superior temporal sulcus, middle temporal gyrus +, etc.) [22, 39, 80] largely independent of general cognitive processes [72, 92, 97], including intelligence quotient (IQ) [29]. In contrast, BM performance often correlates positively with IQ in autistic individuals [29], suggesting that they may rely on more general cognitive processes [50].

The neural underpinnings of BM perception in ASC-II remain largely unknown, representing a critical gap in our knowledge. ASC-IA individuals often exhibit reduced efficiency or accuracy during BM processing [91] but may demonstrate the ability to discriminate BM point-light displays from scrambled point-light displays on par with TDCs on some tasks [27], potentially via compensatory neural strategies or heightened attentional deployment that can normalize activation patterns [53]. Nonetheless, whether these mechanisms are available or effective in ASC-II is unknown. To address these gaps, this study used functional MRI during a passive BM viewing task in TDC, ASC-IA, and ASC-II to investigate: 1) Group-average differences across TDC, ASC-IA, and ASC-II groups; 2) if idiosyncrasy in whole-brain BM activation patterns differs across the TDC, ASC-IA, and ASC-II groups; and 3) if the degree of brain idiosyncrasy during BM perception is associated with core autistic traits and related cognitive and behavioural characteristics transdiagnostically across all participants. We hypothesized our ASC cohort to exhibit hypoactivation in BM perception-associated regions (e.g. posterior superior temporal sulcus, middle temporal gyrus, inferior parietal lobule) compared to TDC in accordance with previous literature [7, 30, 50, 91, 99, 100], with the effect being stronger in ASC-II than ASC-IA due to the negative association between IQ and BM perception task performance. Given the pronounced heterogeneity in ASC and the potential reliance on variable, generalized cognitive resources, we hypothesized that brain responses to BM might be particularly idiosyncratic (i.e., variable across individuals) in ASC, an effect potentially amplified in the ASC-II group. If ASC is indeed associated with idiosyncratic brain activity, we would also expect idiosyncrasy to correlate with the expression of autism-relevant cognitive/behavioural traits.

## Methods

### Participants

Participant recruitment and experimental procedures were conducted in accordance with the Declaration of Helsinki and were approved by the Research Ethics Committee of the National Taiwan University Hospital, Taiwan (#201512238RINC; Clinical trial number: not applicable). Written informed consent was obtained from all participants capable of providing consent and from parents/legal guardians for all participants under 18 or who were unable to consent. Assent was obtained from participants unable to provide informed consent themselves, supplemented by consent from a substitute decision-maker.

One hundred twenty-five participants (aged 8–30 years) were initially recruited between December 2016 and August 2019. Three individuals were excluded as they did not proceed with MRI scanning. The remaining 122 participants included 83 autistic individuals referred from several psychiatric outpatient clinics in Taiwan and 39 TDC participants recruited from the community and matched for age and socioeconomic background. Autism spectrum disorder diagnosis was established by experienced child psychiatrists according to DSM-5 criteria and confirmed upon enrollment by the senior author (a double-board child psychiatrist) using the Autism Diagnostic Observation Schedule-Second Edition (ADOS-2; appropriate module selected based on age/language level) [13, 15, 58] and the Autism Diagnostic Interview-Revised (ADI-R; Diagnostic Algorithm used for confirmation) [45, 59]. ADOS-2 Calibrated Severity Scores (CSS) [38, 46] were used in analyses where relevant. Parents of all participants were interviewed by the senior author using the Kiddie-Schedule for Affective Disorders and Schizophrenia-Epidemiological Version (K-SADS-E) for DSM-5 [3, 16, 25] to screen for major psychiatric conditions. TDC participants were confirmed to have no personal or first-degree-relative history of current or past DSM-5 or other major neuropsychiatric diagnoses via this interview. Exclusion criteria included major medical illness or neuropsychiatric conditions (e.g., bipolar, psychotic, or substance use disorders, current suicidality ideation), significant head trauma, active seizures, known genetic syndromes, or pregnancy. Participants with co-occurring ADHD or adjustment disorder were included. Participants taking stimulant medication withheld it for 24 h prior to scanning. Table S1 in Additional File presents details of medication use and co-occurring psychiatric conditions in autistic individuals. All participants underwent standard MRI safety screening prior to the scan, including checks for metallic implants, pacemakers, and claustrophobia.

Cognitive function was assessed using the age-appropriate Wechsler Intelligence Scale—4th Edition [WAIS-IV [96] or WISC-IV [95]], with full-scale IQ (FIQ) used for the primary purpose of grouping participants. Additionally, given that standard intelligence tests with verbal components can underestimate cognitive capacity in individuals with significant language or performance impairments [18], all participants were also assessed with the Leiter International Performance Scale-Revised (Leiter-R) [71] to obtain a standardized measure of non-verbal IQ (NVIQ). For the subsequent transdiagnostic brain-behaviour correlation analyses, the Leiter-R NVIQ was selected as the definitive measure of cognitive ability. This choice was made for two key reasons. First, it provided a consistent metric of non-verbal intelligence that could be validly obtained from the entire sample, including individuals with minimally verbal status or severe intellectual disability who were unable to complete all subtests of the Wechsler scales. Previous literature indicates that Wechsler scales often underestimate intelligence in autistic individuals [14, 18]—particularly those with language impairments—whereas the Leiter-R provides a valid assessment that correlates with adaptive function [62]. Second, using the Leiter-R allowed for a more direct examination of the relationship between non-verbal cognitive abilities and the neural processing of the non-verbal BM task, thereby minimizing potential confounds from verbal intelligence. To validate this choice, we assessed the correlation between Leiter-R NVIQ and Wechsler Full-Scale IQ in the subset of participants who were able to complete both assessments (*n* = 89). We found a strong, significant correlation (*r* = 0.79, *p* < 2*10^–16^), supporting the validity of Leiter-R NVIQ as a proxy for general cognitive ability in this sample.

The whole ASC group (ASC-Whole) was divided into intellectually able (ASC-IA) and intellectually impaired (ASC-II) subgroups. Participants who scored < 85 on Wechsler FIQ or VABS-ABC were assigned to the ASC-II subgroup (*n* = 46 initially), whereas participants with ≥ 85 on both tests were assigned to the ASC-IA subgroup (*n* = 37 initially). This inclusive definition—using an FIQ cutoff approximately 1 SD below the mean and incorporating adaptive function—aimed to capture individuals with significant functional challenges, as autistic children with IQs in the borderline range (i.e., 70–84) often have similar developmental outcomes to children with co-occurring autism and intellectual disability [67]. We chose to use the terminology of “intellectually able/impaired” instead of “high/low functioning” to avoid potentially inaccurate and prejudicial implications of the latter terms [2].

### Parent-rated assessments

Participants were assessed using parent-rated scales including the Social Responsiveness Scales (SRS) [17, 34] for autistic traits; the Vineland Adaptive Behavior Scales—Adaptive Behavior Composite (VABS-ABC) [60, 82] for adaptive function (Adaptive Behavior Composite [ABC] used for analyses herein); Behavior Rating Inventory of Executive Function—Global Executive Composite (BRIEF-GEC) [36] for daily life executive function; Swanson, Nolan, and Pelham ADHD Rating Scale (SNAP-IV) [33, 85] for ADHD symptoms, Short Sensory Profile (SSP) [61] for sensory processing difficulties, and Repetitive Behavior Scale-Revised (RBS-R) [9] for repetitive behaviours.

### MRI acquisition

Imaging data was collected on a Siemens MAGNETOM Prisma 3-T MRI with a 64-channel phased-array head/neck coil. A high-resolution MPRAGE T1-weighted sequence was acquired sagittally (repetition time [TR] = 2000 ms; echo time [TE] = 2.43 ms; inversion time [TI] = 920 ms; flip angle = 9°; matrix = 256 × 256; FOV = 230 mm; 192 slices; voxel size = 0.9 mm isotropic). A multiband multi-echo (MBME) gradient-echo EPI sequence was used for the BM task fMRI acquisition (TR = 1320 ms; TEs = 14.2/35.25/56.3 ms; FOV = 210 mm, matrix = 80 × 80; 48 slices acquired in interleaved order with MB factor 3; voxel size = 2.6 mm isotropic; flip angle = 60°; partial Fourier = 6/8; in-plane acceleration = 2). The broader scanning session for this cohort also included resting-state fMRI (rsfMRI) and diffusion MRI (dMRI) sequences, acquired alongside the T1-weighted and BM task fMRI. Analyses of the dMRI data from these participants have been published elsewhere [101]. Following MRI scanning, participants who exhibited an inability to complete the BM fMRI (which was scanned after T1-weighted image, resting-state fMRI, and diffusion MRI) or exhibited excessive head motion during the scan (based on mean framewise displacement [FD] > 2 SD above the iterative sample mean) were excluded from the analyses [98].

### Task design

Participants performed a passive viewing task involving silent point-light display videos during fMRI scanning. The stimuli [50] consisted of BM point-light displays and scrambled motion control point-light displays. BM point-light displays depicted a male actor performing recognizable actions (e.g., pat-a-cake), derived from motion-capture data. Scrambled motion point-light displays were generated by spatially randomizing the starting position of each dot from the corresponding BM point-light display while preserving individual dot trajectories, thus matching local motion properties but lacking coherent biological form. Each video was 24 seconds long, with the task consisting of 12 consecutive videos presented without an inter-stimulus interval. Six biological motion clips and six scrambled motion clips were presented once each in an alternating-block design (time per block, 24s). The experiment began and ended with a 20-s fixation period (total time, 328 s) [50]. Stimuli were displayed using E-Prime 2.0 software (Psychology Software Tools, Inc.) and back-projected onto a screen visible via a mirror mounted on the head coil. Participants were instructed to remain still, keep their eyes open, and attentively watch the videos, but no response was required. This passive design was chosen to minimize cognitive demands and performance confounds, thereby enhancing suitability for participants across a range of intellectual abilities. Participants' alertness was verified by their prompt responses to questions immediately prior to and following the BM fMRI scan.

### Data preprocessing

fMRI image preprocessing utilized AFNI (v24.1.8), including steps for slice timing, realignment, co-registration to the T1 image, and normalization through nonlinear warping to the MNI space (voxel size 2.6 mm isotropic). The normalization utilized a DARTEL (Diffeomorphic Anatomical Registration Through Exponentiated Lie Algebra) template [4]. The normalized data were then denoised using TE-Dependent Analysis (TEDANA) [24] and multi-echo independent component analysis (ME-ICA v3.2) [54] to remove non-BOLD components from the fMRI data. T1c global signal regression was then applied to the data to remove spatially diffuse noise. ME-ICA's robust denoising allowed us to skip spatial smoothing, aiming to preserve interindividual spatial variability (Kundu et al., 2017) [65]. See Additional File: Supplementary Methods for details on multi-echo fMRI denoising.

### fMRI data analysis

Statistical analyses were performed using Statistical Parametric Mapping (SPM12; Wellcome Trust Centre for Neuroimaging; https://www.fil.ion.ucl.ac.uk/spm/software/spm12/) [79] and Statistical Nonparametric Mapping (SnPM13,http://nisox.org/Software/SnPM13/) for permutation-based inference. First-level general linear models (GLMs) were estimated for each participant. These models included boxcar regressors representing the timing of BM and scrambled motion (SCR) blocks, convolved with the canonical hemodynamic response function. Nuisance regressors comprised six motion parameters derived from realignment and the mean FD for the run. Contrasts were defined to estimate activation specifically for BM > SCR and SCR > BM. These first-level contrast maps were carried forward to group-level analyses conducted within SnPM using 10,000 permutations. One-sample t-tests assessed the mean activation pattern for the BM > SCR contrast within each group (TDC, ASC-Whole, ASC-IA, and ASC-II) separately. To identify differences between groups, two-sample t-tests directly compared the BM > SCR contrast maps (TDC vs. ASC-Whole, TDC vs. ASC-IA, TDC vs. ASC-II, ASC-IA vs. ASC-II). All group-level analyses incorporated assigned sex at birth, age, and mean FD as nuisance regressors. To correct for multiple comparisons across the whole brain volume, resulting group activation maps were generated using threshold-free cluster enhancement (TFCE) [79], as implemented in SnPM13, with statistical significance determined at a cluster-level false discovery rate controlled at 0.05.

### Idiosyncrasy

Brain idiosyncrasy was quantified as “variability” [31, 32, 42] for each participant, reflecting the distinctiveness of their individual BM > SCR activation pattern relative to the entire sample (i.e., “whole-sample variability” [Variability_Whole_] across autistic and TDC participants). Each participant’s BM > SCR t-statistic map was parcellated into 284 regions of interest (ROIs) by combining the Schaefer 200-parcel atlas [74], the Melbourne Subcortex atlas (Scale III, 50 regions) [90], and the SUIT Cerebellum atlas (34 regions) [21], generating a 1 × 284 vector that summarized the whole-brain activation pattern for each participant. R-4.2.0 was used to compile these vectors into a participant × ROI matrix, and to perform all subsequent statistical analyses (ANCOVA, GAM). The correlational distance (defined as 1–Pearson’s *r*) was computed between the activation vectors of all participant pairs, where lower distances indicate greater pattern similarity. An individual’s Variability_Whole_ was defined as their average correlational distance to all other participants in the sample; higher values thus represent a more idiosyncratic brain pattern. Recognizing that the choice of reference group impacts the interpretation of idiosyncrasy, we additionally calculated variability referenced to an individual’s own diagnostic group (i.e., either TDC or ASC; “within-group brain variability,” Variability_Within_) and to the TDC group (mean distance to TDC participants; “TDC reference brain variability,” Variability_TDC_) in supplementary analyses to provide a more nuanced perspective on individual brain differences. Group differences (TDC vs. ASC-IA vs. ASC-II) in the variability metrics (Variability_Whole_ as the primary, Variability_Within_ and Variability_TDC_ as the supplementary) were assessed using a One-way ANCOVA with age, sex, and mean FD as covariates, followed by post hoc pairwise ANCOVA tests with Bonferroni correction for pairwise comparisons. Effect sizes were reported using partial eta squared (η_p_^2^) and Cohen’s d. To assess potential effects of co-occuring ADHD on brain idiosyncrasy, we performed supplementary one-way ANCOVA analyses on the ASC-Whole, ASC-IA and ASC-II groups divided by presence of ADHD. 

### Brain behaviour correlations

We examined transdiagnostic relationships between Variability_Whole_ and the assessed cognitive and behavioural measures across the entire sample using generalized additive models (GAMs). The distribution of age, mean FD, and each cognitive/behavioural measure was first checked for normality using the Kolmogorov–Smirnov test [63]. Measures approximating a normal distribution (ADOS-2 CSS, BRIEF-GEC, SNAP-IV Total, VABS-ABC), were treated as linear terms, predicting Variability_Whole_ from the behavioural score while controlling for sex, age, and mean FD. The other four measures deviating from normality (SRS Total, RBS-R, SSP Total, NVIQ) were treated as smooth terms, with the same covariates to allow for potentially nonlinear associations. Age approximated a normal distribution while mean FD did not, and were thus treated as linear and smooth in all GAMs respectively. Supplementary analyses were also performed for Variability_Within_ and Variability_TDC_. The Benjamini–Hochberg False Discovery Rate (FDR) [5] was used to correct for multiple tests (across the eight primary behavioral predictor terms), applying a significance threshold of q < 0.05.

### Sensitivity analysis

Finally, to rule out potential confounding effects of sex given the absence of female participants in the ASC-II group, we performed a complete sensitivity analysis (group differences and transdiagnostic brain-behaviour correlations) using male participants only.

## Results

### Demographics

The final sample included in this study comprised 47 autistic (28 ASC-IA, 19 ASC-II) and 33 TDC participants. TDC, ASC-IA, and ASC-II had comparable distributions of age and framewise displacement (all ps > 0.05; Table 1). The sex ratio differed significantly between TDC and ASC-II (*p* < 0.05), and the medication and comorbidity ratios differed between TDC and the other two groups (*p* < 0.001). Distribution of cognitive and behavioural test scores varied between groups across all eight tested dimensions (*p* = 0.0019 for ADOS-2 CSS, *p* < 0.001 for others). A complete list of our sample’s demographic and clinical characteristics in addition to pairwise differences, as determined by post-hoc testing, is provided in Table 1.

**Table 1:** Demographic data and clinical features of participants

	Typically developing control (TDC) *n* = *33*		Intellectually able ASC (ASC-IA) *n* = *28*		Intellectually impaired ASC (ASC-II) *n* = *19*		*P* value	Post-hoc test
	Mean	SD	Mean	SD	Mean	SD		
Sex (M:F)	25:8		25:3		19:0		0.043^a^	ASC-II ≠ TDC, ASC-II = ASC-IA, ASC-IA = TDC^c^
Age (years)	17.5	5.67	16.1	5.48	17.5	6.64	0.59^b^	-
FD	0.0705	0.0311	0.0899	0.0471	0.0942	0.0421	0.067^b^	-
Medication (Y:N)	0:33		16:12		9:10		< 0.001^a^	ASC-II, ASC-IA ≠ TDC^c^
Comorbidity (Y:N)	0:33		18:10		12:7		< 0.001^a^	ASC-II, ASC-IA ≠ TDC^c^
Cognition and behaviour								
SNAP-IV Total	4.2	5.2	20.2	9.4	24.9	12.4	< 0.001^b^	ASC-II, ASC-IA > TDC^d^
SRS Total	18.0	10.7	84.7	28.6	99.6	22.5	< 0.001^b^	ASC-II, ASC-IA > TDC^d^
RBS-R Total	3.3	5.4	26.3	19.6	27.4	20.4	< 0.001^b^	ASC-II, ASC-IA > TDC^d^
BREIF-GEC	90.2	16.7	149.5	25.9	156.8	25.4	< 0.001^b^	ASC-II, ASC-IA > TDC^d^
VABS-ABC	113.5	15.6	91.1	14.5	67.0	10.3	< 0.001^b^	ASC-II < ASC-IA < TDC^d^
SSP Total	185.0	6.0	162.3	22.4	153.8	20.9	< 0.001^b^	ASC-II, ASC-IA < TDC^d^
ADOS-2 CSS	N/A	N/A	4.89	2.02	6.89	2.05	0.0019^b^	ASC-II < ASC-IA^b^
NVIQ	122.3	10.3	117.2	16.2	79.0	23.7	< 0.001^b^	ASC-II < ASC-IA,TDC^d^

*SNAP-IV* Swanson, Nolan, and Pelham ADHD Rating Scale, *SRS* Social Responsiveness Scales, *RBS-R* Repetitive Behavior Scale–Revised, *BRIEF-GEC* Behavior Rating Inventory of Executive Function—Global Executive Composite, *VABS-ABC* Vineland Adaptive Behavior Scales—Adaptive Behavior Composite, *SSP* Short Sensory Profile, *ADOS-2 CSS* Autism Diagnostic Observation Schedule-2 Calibrated Severity Score, *NVIQ* Nonverbal Full-Scale Intelligence Quotient (Leiter-R)

^a^Chi-square

^b^ANOVA

^c^Pairwise Chi-square tests

^d^Tukey-Kramer Test

### Group-average activation (BM > SCR contrast)

**Fig. 1:**
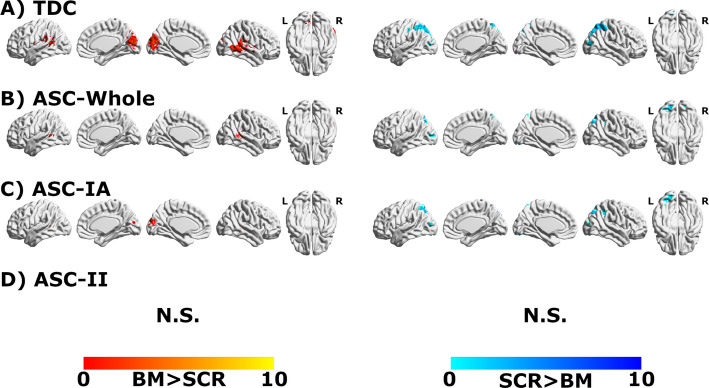
Group-average brain activation for Biological Motion (BM)>Scrambled Motion (SCR) in (**A**) typically developing controls (TDC), **B** autism spectrum condition (ASC)-Whole, and **C** ASC-Intellectually Able (IA). No consistent BM-dependent response was observed in the (**D**) ASC-Intellectual Impairment (II) subgroup

One-sample t-tests (p_FDR_ < 0.05, cluster-corrected via TFCE) revealed significant activation clusters in the TDC, ASC-Whole, and ASC-IA groups. Consistent with expectations, these clusters included bilateral pSTS and cuneus (Fig. 1a-c). In marked contrast, no significant clusters for the BM > SCR contrast were observed in the ASC-II group at the same statistical threshold, indicating the absence of a detectable, consistent group-average BM-specific activation pattern in these individuals (Fig. 1d).

### Between-group differences in activation (BM > SCR contrast)

**Fig. 2:**
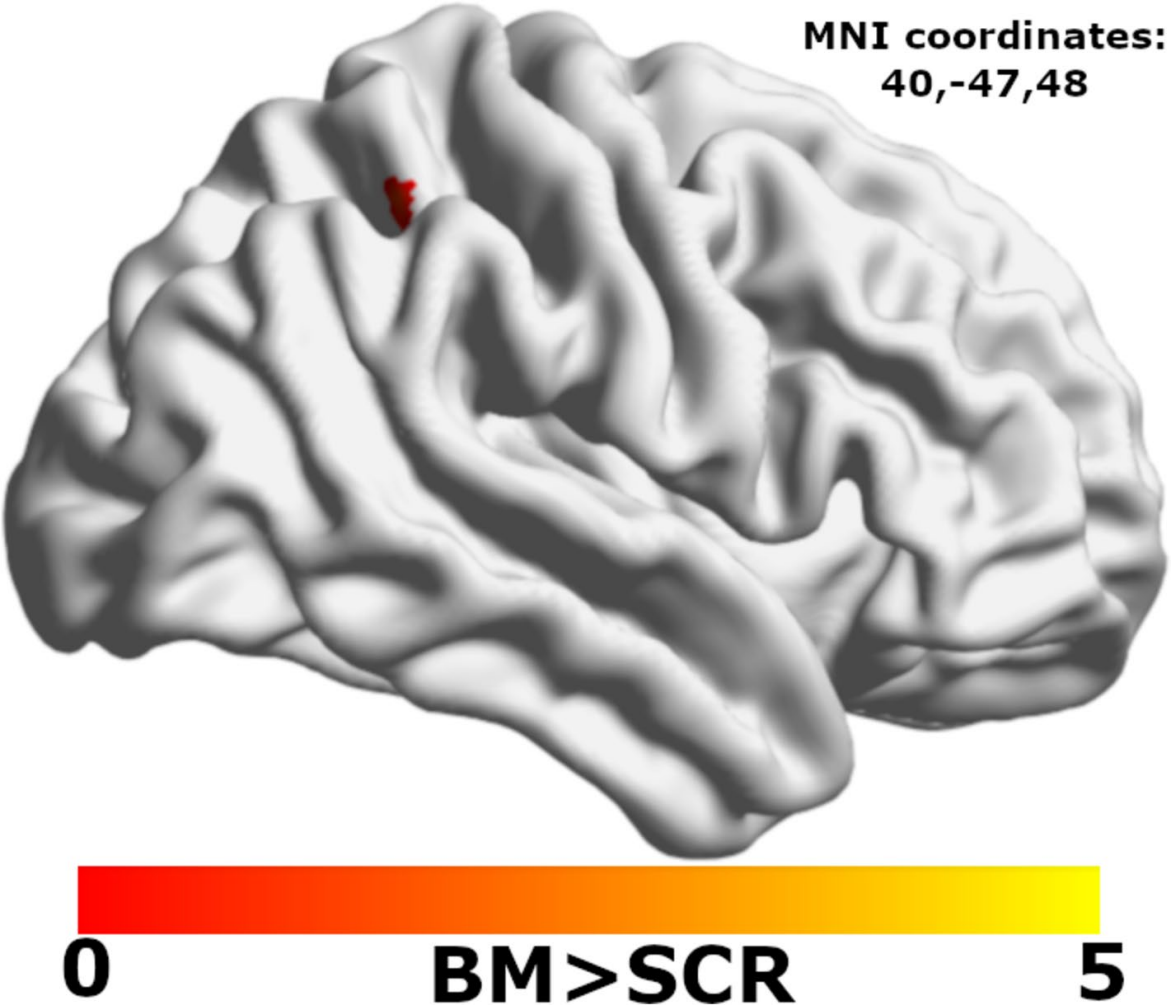
Group difference in Biological Motion (BM)-evoked activation (BM > Scrambled Motion [SCR]) of the right intraparietal sulcus in Autism Spectrum Condition-Intellectual Impairment (ASC-II) compared to ASC-Intellectually Able (IA)

Two-sample t-tests (p_FDR_ < 0.05, cluster-corrected via TFCE), controlling for age, sex, and mean FD, yielded only one significant finding: the ASC-II group exhibited significantly greater activation in the right intraparietal sulcus (rIPS) compared to ASC-IA group (k = 13 voxels, peak MNI coordinates = 40, −47, 48; Fig. 2). No other significant group differences survived correction.

### Brain idiosyncrasy (Variability_Whole_) across groups

**Fig. 3:**
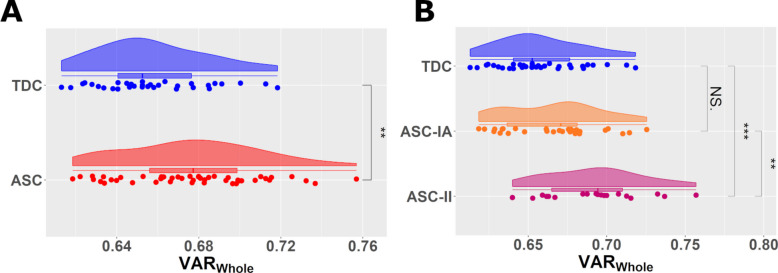
Variability (VAR)_Whole_ across diagnostic groups. Raincloud plots illustrate the distribution of Variability_Whole_ across participants. ANCOVA (controlling for age, sex, and mean framewise displacement) was used to assess group effects on variability. Autism spectrum condition (ASC)-Whole (*n* = 47, all autistic participants regardless of intelligence) exhibited greater Variability_Whole_ than typically developing control (TDC) (*n* = 33) (**A**, **p_FWE_ < 0.01). Significant group effects were also observed when stratifying the ASC group into ASC-Intellectually Able (IA) and ASC-Intellectual Impairment (II) (**B**). *Post-hoc* pairwise ANCOVA tests with Bonferroni correction showed that ASC-II exhibited greater Variability_Whole_ than both ASC-IA (*p_FWE_ < 0.05) and TDC (***p_FWE_ < 0.001), but ASC-IA did not differ significantly from TDC (p_FWE_ > 0.05). NS.: Not significant

We investigated whether brain idiosyncrasy differed across the groups using a one-way ANCOVA, controlling for age, sex, and mean FD. Using two groups (TDC vs ASC-Whole) in the model (Fig. 3a) revealed a significant main effect of diagnostic group (F = 8.146, *p* = 0.0056, η_p_^2^ = 0.050, d = 0.63) on Variability_Whole_. A significant age-effect (F = 4.962, *p* = 0.029, η_p_^2^ = 0.034) on Variability_Whole_ was also found, suggesting lower variability is associated with increasing age. When stratifying the autistic group based on IQ in a three-group model (TDC vs ASC-IA vs ASC-II; Fig. 3b), group (F = 10.005, *p* < 0.001, η_p_^2^ = 0.173) and age (F = 7.178, *p* = 0.009, η_p_^2^ = 0.055) effects remained significant. *Post-hoc* testing using pairwise ANCOVA tests with Bonferroni correction indicated that Variability_Whole_ in the ASC-II group was significantly higher than in both the TDC (p_FWE_ < 0.001, d = 1.24) and ASC-IA (p_FWE_ = 0.010, d = 0.89) groups. No significant differences in Variability_Whole_ were observed between the TDC and ASC-IA groups (p_FWE_ = 0.75). Additional File: Fig. S1 presents the distribution of Variability_Within_ and Variability_TDC_ across our sample.

### Brain idiosyncrasy and ADHD

No significant differences in Variability_Whole_ (F = 0.078, p = 0.78, η_p_^2^= 3.2*10^-4^; Fig. S2A), Variability_Within_ (F = 0.23, p = 0.64, η_p_^2^ = 2.2 * 10^-4^; Fig. S2C), and Variability_TDC_ (F = 0.010 , p = 0.92, η_p_^2^ =1.9 * 10^-3^; Fig. S2E), were observed between autistic participants (no TDCs had ADHD) with (ASC + ADHD) and without ADHD (ASC - ADHD). We additionally stratified the ASC + ADHD and ASC - ADHD groups based on intellectual capacity [ASC-IA + ADHD (n = 11) vs. ASC-IA - ADHD (n = 17) vs. ASC-II + ADHD (n = 8) vs. ASC-II - ADHD (n = 11)] and found a significant group effect on Variability_Whole_ (F = 3.17, p = 0.035, η_p_^2^ = 0.18; Fig. S2B) and Variability_Within_ (F = 3.52, p = 0.024, η_p_^2^ = 0.20; Fig. S2D), but not Variability_TDC_ (F = 2.61, p = 0.065, η_p_^2^ = 0.16, Fig. S2F). However, no pairwise group differences remained significant following post-hoc pairwise ANCOVAs with Bonferroni correction (all p_FWE_ > 0.05). Additional File: Fig. S2 presents the distribution of all three Variability metrics across autistic participants with and without ADHD.

### Transdiagnostic correlations between Variability_Whole_ and cognitive/behavioural traits

**Fig. 4:**
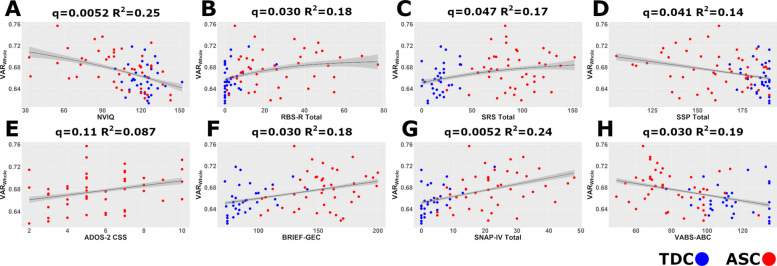
Transdiagnostic associations between brain idiosyncrasy (Variability [VAR]_Whole_) and cognitive/behavioural measures. Scatterplots depict relationships across all participants, controlling for age, sex, and mean framewise displacement. Significant linear associations (false discovery rate [FDR] q < 0.05) were found for Behavior Rating Inventory of Executive Function—Global Executive Composite (BRIEF-GEC) (**F**), Swanson, Nolan, and Pelham ADHD Rating Scale (SNAP-IV) Total (**G**), and Vineland Adaptive Behavior Scales—Adaptive Behavior Composite (VABS-ABC) (**H**). Significant nonlinear associations (generalized additive models, FDR q < 0.05) were found for non-verbal full-scale intelligence quotient (NVIQ) (**A**), Repetitive Behavior Scale-Revised (RBS-R) Total (**B**), Social Responsiveness Scale (SRS) Total (**C**), and Short Sensory Profile (SSP) Total (**D**) (lines show model fits). Autism Diagnostic Observation Schedule-Second Edition (ADOS-2) Calibrated Severity Score (CSS) (**E**) was not significantly associated. Shaded areas represent 95% confidence intervals. Notably, higher SSP scores represent fewer sensory symptoms

Across the whole sample (Fig. 4; see Additional File: Table S2 for full model statistics), after controlling for age, sex, and mean FD, higher idiosyncrasy (Variability_Whole_) was significantly linearly associated with greater parent-reported ADHD symptoms (SNAP-IV Total: q = 0.005, R^2^ = 0.242; Fig. 4g), greater executive dysfunction in daily life (BRIEF-GEC: q = 0.030, R^2^ = 0.183; Fig. 4f), and lower adaptive functioning (VABS-ABC: q = 0.030, R^2^ = 0.189; Fig. 4h). No significant linear relationship was identified between Variability_Whole_ and ADOS-2 CSS (q = 0.113; Fig. 4e). Significant nonlinear associations, also controlling for covariates, were found (Table S2 in Additional File): higher Variability_Whole_ was associated with higher parent-reported social difficulties (SRS Total: q = 0.047; Fig. 4c), repetitive behaviours (RBS-R: q = 0.030; Fig. 4b) and sensory processing difficulties (SSP: q = 0.041; Fig. 4d). Higher idiosyncrasy was also associated with lower NVIQ (Leiter-R: q = 0.0052; Fig. 4a). The relationships between cognitive/behavioural variables and the supplementary variability metrics (Variability_Within_/Variability_TDC_) followed a similar pattern of directionality and significance to the primary metric (Variability_Whole_). Notably, for the SSP correlation, while Variability_Within_ correlations remained statistically significant (q < 0.001), the associations with Variability_TDC_ were generally weaker, only reaching marginal significance (q = 0.060). Fig. S3-S4 present the full set of associations between Variability_Within_ and Variability_TDC_ with these cognitive and behavioural metrics, respectively. Additionally, Fig. S5-S7 present these correlations for all three variability metrics when modelling all brain-behaviour associations as linear.

### Sensitivity analysis (Male-Only Sample)

To address potential confounds arising from the sex imbalance in the ASC-II group, we performed a sensitivity analysis excluding all female participants (resulting sample: 25 TDC, 25 ASC-IA, 19 ASC-II). The pattern of group differences in the male-only sample was identical to the whole-sample analysis (see Additional File: Table S3). The male ASC-II group exhibited significantly higher Variability_Whole_ compared to male TDCs and male ASC-IA participants. Consistent with the main analysis, the male ASC-IA and male TDC groups did not differ significantly regarding whole-sample variability. The transdiagnostic associations observed in the whole sample were also replicated in the male-only cohort (see Additional File: Table S4). Higher Variability_Whole_ remained significantly associated with lower non-verbal IQ and lower adaptive function (VABS-ABC). Furthermore, significant positive associations were maintained between idiosyncrasy and symptom severity across social (SRS), repetitive behavior (RBS-R), sensory (SSP), executive function (BRIEF), and ADHD (SNAP-IV) domains. This confirms that the link between brain idiosyncrasy and clinical phenotype is robust to sex effects.

## Discussion

Addressing critical gaps in autism neuroimaging, this study is among the first to investigate the neural correlates of BM perception in ASC-II and ASC-IA. We report three primary findings: 1) the ASC-II group lacked a consistent group-average activation pattern seen in other groups; 2) this was explained by significantly elevated brain idiosyncrasy in the ASC-II group; and 3) this idiosyncrasy was robustly associated with transdiagnostic cognitive and clinical features. These results challenge the sufficiency of group-average approaches and highlight brain idiosyncrasy as a critical dimension linking brain function to clinical phenotypes across the full autism spectrum.

### Neural correlates of BM perception: averages mask clinically relevant idiosyncrasy

Consistent with extensive prior work [44, 80, 94], the TDC, ASC-Whole, and ASC-IA groups showed reliable activation in canonical BM processing regions, including the pSTS and cuneus, during passive viewing. The striking absence of any detectable group-average BM > SCR activation in the ASC-II group, despite using identical methods, aligns strongly with our finding of significantly elevated brain idiosyncrasy (primarily assessed via Variability_Whole_; see Supplementary Material for similar trends in other metrics) in this specific subgroup. This suggests that the lack of a consistent group map in ASC-II is not necessarily due to a consistent absence of BM-related processing in individuals but rather stems from high idiosyncrasy in the specific brain patterns elicited, which average out at the group level. This interpretation supports the hypothesis, prompted by the IQ-dependence of BM performance in ASC [29], that typical specialized BM circuitry [39, 80] may be disrupted or less consistently engaged in ASC-II, leading to the recruitment of more variable brain regions across individuals.

Surprisingly, our conventional group comparisons yielded minimal significant differences in mean activation between the TDC and ASC groups, a finding also noted in some prior work [1, 47], though contrasting with other studies reporting widespread hypoactivation in ASC during BM tasks [7, 30, 50, 99, 100]. The only significant group difference in mean activation was greater rIPS engagement in ASC-II versus ASC-IA participants. The rIPS is implicated in processing visual motion and potentially discerning animacy [77], suggesting its heightened engagement in ASC-II might reflect altered attentional processing or a compensatory strategy, particularly given that task performance tends to decrease with lower IQ in ASC [29]. The general absence of significant TDC versus ASC group differences in mean activation might partially reflect rigorous methodology, including advanced ME-ICA denoising [19, 26, 35, 54, 84] and sensitive statistical thresholding [79], which minimize spurious findings. However, the crucial insight emerges from the combination of these minimal *average* differences with the markedly increased *individual* idiosyncrasy, especially in ASC-II. Consistent with prior work using idiosyncrasy [31, 32, 42, 76, 78, 86], our study provides empirical evidence to argue that group averages provide an incomplete, potentially misleading picture of processing differences in clinical samples. The most critical neurobiological variation appears to lie at the individual level, particularly for the ASC-II group.

### ASC-II exhibits markedly elevated and uniquely profiled brain idiosyncrasy in BM processing

A central finding of this study, which explains the lack of a group-average map discussed above, is the significantly higher brain idiosyncrasy during BM perception observed specifically in the ASC-II group, compared to both ASC-IA and TDC groups. While ASC-IA did not differ from TDCs on Variability_Whole_ or Variability_TDC_, they exhibited elevated within-group heterogeneity (Variability_Within_) compared to TDC (See Fig. S1), suggesting subtle differences even in this subgroup. This overall pattern contributes to a growing literature suggesting that neurobiological idiosyncrasy is a key characteristic of autism [6, 10, 12, 20, 40, 42, 66, 69, 93]. Previous work has documented increased idiosyncrasy in autistic individuals (often primarily ASC-IA samples) across various domains, including functional connectivity [83], task-based brain responses [10, 11, 41, 42, 68, 73, 75], white matter microstructure [93], and time-coordinated social behaviours [8]. Our results extend this concept of autistic idiosyncrasy to BM perception, intriguingly revealing a specific profile where the most pronounced elevation in overall idiosyncrasy (Variability_Whole_) is linked to intellectual impairment.

The specificity of elevated idiosyncrasy to the ASC-II aligns compellingly with the hypothesis linking intellectual function to BM processing uniquely in ASC. As discussed, BM perception in typical development seems reliant on specialized mechanisms that are largely independent of IQ [72, 92]. If this specialization is altered in ASC (perhaps due to disruptions in underlying circuitry), reliance may shift towards more general cognitive resources, thereby making the process IQ-dependent [29]. We propose that individuals with greater cognitive resources (ASC-IA and TDC) may more effectively recruit and constrain brain activity towards a relatively consistent pattern, reflecting either intact specialization or successful compensatory/attentional strategies [53], resulting in lower idiosyncrasy. The elevated idiosyncrasy specific to ASC-II may arise from an interplay between potentially altered network specialization for BM, compensatory reliance on general cognitive functions, and the modulating influence of intellectual capacity, especially attentional and executive control abilities [28]. The significant negative association we observed between NVIQ and Variability across the entire sample provides strong transdiagnostic support for this interpretation, linking lower cognitive ability with more idiosyncratic brain patterns during BM perception.

### Brain idiosyncrasy transdiagnostically correlates with cognitive abilities and clinical features

Beyond group differences, our findings reveal robust transdiagnostic associations between the degree of brain idiosyncrasy (primarily Variability_Whole_; see Supplementary Material Figs. S3-S7 for similar patterns with other metrics) and a wide array of clinically relevant characteristics. Higher idiosyncrasy was significantly linked to lower non-verbal IQ (NVIQ) and poorer adaptive functioning (VABS-ABC), suggesting that more atypical individual brain patterns relate to broad difficulties in cognitive abilities and real-world skills. Furthermore, higher idiosyncrasy correlated with greater parent-reported difficulties in domains central to autism, including social responsiveness (SRS Total), repetitive behaviours (RBS-R Total), and sensory processing (SSP Total), as well as co-occurring challenges like ADHD symptoms (SNAP-IV Total) and executive dysfunction (BRIEF-GEC). The dimensional correlation between ADHD symptoms and brain idiosyncrasy during a BM perception task is particularly notable, despite no significant categorical differences among autistic subgroups with and without ADHD (Fig. S2). Attention is a critical modulator of biological motion processing [87]. We speculate that the characteristic attentional variability or “lapses” associated with ADHD symptoms may lead to inconsistent neural engagement with the stimuli. In a passive viewing task, fluctuations in sustained attention could result in highly variable neural recruitment within individuals across the scan duration. This unstable internal processing likely manifests as a more unique, idiosyncratic activation pattern when averaged over the scan, thereby increasing inter-individual variability compared to those with more stable attentional focus. This adds to a growing body of literature linking co-occurring ADHD traits with alterations in social perceptual processing [89]. These results suggest that brain idiosyncrasy during even a specific task like passive BM viewing may reflect broader principles of brain organization or processing efficiency that impact multiple functional domains. This aligns with previous work linking brain idiosyncrasy to cognitive performance, including social comprehension [12], generalized and social cognition [78], and executive function [42]. The widespread associations could arise if less constrained, more variable brain activity for BM encroaches upon or interferes with other networks, or if idiosyncrasy itself is a marker of less efficient or less specialized cognitive processing across systems. The lack of a significant association with ADOS-2 CSS (q = 0.113), despite correlations with other autism-relevant measures (SRS, RBS-R, SSP), is noteworthy. We attempted to address the potential impact of the data distribution by modelling ADOS-2 CSS as an ordinal variable; however, this approach similarly failed to reveal a significant relationship with brain idiosyncrasy. This may reflect measurement limitations of the ADOS-2 CSS or suggest that passive brain idiosyncrasy relates more strongly to dimensional questionnaire traits than to observer-rated severity in a specific context.

The observed transdiagnostic correlation between higher brain idiosyncrasy and greater clinical challenges requires careful interpretation. While brain diversity is evolutionarily adaptive [64, 70], our findings support a continuum model: idiosyncrasy is a fundamental dimension of brain organization where clinical relevance emerges at its extremes [37] rather than reflecting an intrinsically pathological trait.

### Limitations

Several limitations should be considered. First, the cross-sectional correlational nature of our brain-behaviour findings precludes causal inferences. Future work using brain stimulation methods could probe causal links. Second, our passive viewing task did not include a behavioural measure of BM perception accuracy or reaction time, limiting our ability to directly link brain idiosyncrasy to task performance itself. Third, while we verified participant alertness pre- and post-scan, we lacked concurrent eye-tracking to confirm continuous visual attention to the stimuli throughout the task [51, 81]. Fourth, the sample has a significant sex bias towards males, reflecting known challenges in recruiting autistic females. However, our sensitivity analysis excluding females (Tables S3 & S4) confirmed that both the elevated idiosyncrasy in ASC-II and the transdiagnostic correlations hold true in a male-only cohort, suggesting these effects are not driven by sex differences. Fifth, the wide age range of our participants (8–30 years), encompassing childhood, adolescence, and young adulthood, is a further limitation. While age was included as a linear covariate in our analyses, this statistical control may not fully capture the complex, potentially nonlinear neurodevelopmental changes occurring across this broad developmental window, which could influence BM processing, brain idiosyncrasy, and their associations. Sixth, our findings are specific to BM perception; future research should investigate whether elevated brain idiosyncrasy in ASC-II generalizes across different social and non-social tasks. Seventh, although stimulant medications were withheld 24 h prior to scanning, we did not account for potential chronic effects of medication history.

## Conclusion

This study provides novel evidence on the brain processing of biological motion in autism, particularly by including the often-excluded subgroup of individuals with co-occurring intellectual impairment (ASC-II) and focusing on individual-level brain idiosyncrasy. We demonstrated that the ASC-II group lacks a consistent group-average BM activation pattern, exhibits significantly elevated brain idiosyncrasy compared to both ASC-IA and TDC groups, and shows heightened activation only in the rIPS relative to ASC-IA. Crucially, the degree of brain idiosyncrasy during this passive social perception task was strongly associated with core cognitive abilities (nonverbal IQ), adaptive functioning, and multiple clinical domains (autistic traits, executive function, ADHD symptoms) transdiagnostically. Our results underscore the limitations of relying solely on group-average analyses in heterogeneous conditions like autism and highlight brain idiosyncrasy not as mere noise, but as a potentially critical, clinically relevant feature of brain function, especially prominent in ASC-II. These findings compel a shift in autism neuroimaging, advocating for research practices that embrace and quantify idiosyncrasy to achieve a more comprehensive, ecologically valid, and nuanced understanding of the neurobiological underpinnings across the entire autism spectrum. Future work exploring the mechanisms driving idiosyncrasy and its developmental trajectory holds promise for identifying novel markers and potential intervention targets.

## Supplementary Information

Supplementary Material 1.

## Data Availability

The data that support the findings of this study are available from the corresponding author, HYL, upon reasonable request. Public availability of the raw data is restricted by the Research Ethics Committee of the National Taiwan University Hospital in the interest of participant confidentiality. The analysis scripts (R code) used for measuring brain idiosyncrasy and performing statistical analyses are available at https://github.com/chichael-meng/Brain-idiosyncrasy-in-autism.

## References

[1] Alaerts K, Swinnen SP, Wenderoth N. Neural processing of biological motion in autism: an investigation of brain activity and effective connectivity. Sci Rep. 2017;7(1):5612. 10.1038/s41598-017-05786-z. 28717158 10.1038/s41598-017-05786-z PMC5514051

[2] Alvares GA, Bebbington K, Cleary D, Evans K, Glasson EJ, Maybery MT, et al. The misnomer of ‘high functioning autism’: intelligence is an imprecise predictor of functional abilities at diagnosis. Autism. 2020;24(1):221–32. 10.1177/1362361319852831. 31215791 10.1177/1362361319852831

[3] Ambrosini PJ. Historical development and present status of the schedule for affective disorders and schizophrenia for school-age children (K-SADS). J Am Acad Child Adolesc Psychiatry. 2000;39(1):49–58. 10.1097/00004583-200001000-00016. 10638067 10.1097/00004583-200001000-00016

[4] Ashburner J. A fast diffeomorphic image registration algorithm. Neuroimage. 2007;38(1):95–113. 10.1016/j.neuroimage.2007.07.007. 17761438 10.1016/j.neuroimage.2007.07.007

[5] Benjamini Y, Hochberg Y. Controlling the false discovery rate: a practical and powerful approach to multiple testing. J Roy Stat Soc: Ser B (Methodol). 1995;57(1):289–300. 10.1111/j.2517-6161.1995.tb02031.x.

[6] Benkarim O, Paquola C, Park B, Hong S-J, Royer J, de Vos Wael R, et al. Connectivity alterations in autism reflect functional idiosyncrasy. Commun Biol. 2021;4(1):1–15. 10.1038/s42003-021-02572-6. 34526654 10.1038/s42003-021-02572-6 PMC8443598

[7] Björnsdotter M, Wang N, Pelphrey K, Kaiser MD. Evaluation of quantified social perception circuit activity as a neurobiological marker of autism spectrum disorder. JAMA Psychiatr. 2016;73(6):614–21. 10.1001/jamapsychiatry.2016.0219. 10.1001/jamapsychiatry.2016.0219 PMC6475601 27096285

[8] Bloch C, Viswanathan S, Tepest R, Jording M, Falter-Wagner CM, Vogeley K. Differentiated, rather than shared, strategies for time-coordinated action in social and non-social domains in autistic individuals. Cortex. 2023;166:207–32. 10.1016/j.cortex.2023.05.008. 37393703 10.1016/j.cortex.2023.05.008

[9] Bodfish JW, Symons FJ, Parker DE, Lewis MH. Varieties of repetitive behavior in autism: comparisons to mental retardation. J Autism Dev Disord. 2000;30(3):237–43. 10.1023/a:1005596502855. 11055459 10.1023/a:1005596502855

[10] Bolton TAW, Freitas LGA, Jochaut D, Giraud A-L, Van De Ville D. Neural responses in autism during movie watching: inter-individual response variability co-varies with symptomatology. Neuroimage. 2020;216:116571. 10.1016/j.neuroimage.2020.116571. 31987996 10.1016/j.neuroimage.2020.116571

[11] Bolton TAW, Jochaut D, Giraud A-L, Van De Ville D. Brain dynamics in ASD during movie-watching show idiosyncratic functional integration and segregation. Hum Brain Mapp. 2018;39(6):2391–404. 10.1002/hbm.24009. 29504186 10.1002/hbm.24009 PMC5969252

[12] Byrge L, Dubois J, Tyszka JM, Adolphs R, Kennedy DP. Idiosyncratic brain activation patterns are associated with poor social comprehension in autism. J Neurosci. 2015;35(14):5837–50. 10.1523/JNEUROSCI.5182-14.2015. 25855192 10.1523/JNEUROSCI.5182-14.2015 PMC4388936

[13] Chang J-C, Lai M-C, Chien Y-L, Cheng C-Y, Wu Y-Y, Gau SS-F. Psychometric properties of the Mandarin version of the autism diagnostic observation Schedule-Generic. J Formos Med Assoc. 2023;122(7):574–83. 10.1016/j.jfma.2023.01.008. 36732136 10.1016/j.jfma.2023.01.008

[14] Chen F, Planche P, Lemonnier E. Superior nonverbal intelligence in children with high-functioning autism or Asperger’s syndrome. Res Autism Spectr Disord. 2010;4(3):457–60. 10.1016/j.rasd.2009.11.002.

[15] Chen M-H, Huang C-F, Lin Y-S, Chiu Y-N, Gau SS-F, Wu Y-Y. Validation of the Mandarin Chinese version of the Autism Diagnostic Observation Schedule-2 for autism spectrum disorder. Res Autism Spectr Disord. 2023;105:102184. 10.1016/j.rasd.2023.102184.

[16] Chen Y-L, Shen L-J, Gau SS-F. The Mandarin version of the Kiddie-Schedule for Affective Disorders and Schizophrenia-Epidemiological version for DSM-5—a psychometric study. J Formos Med Assoc. 2017;116(9):671–8. 10.1016/j.jfma.2017.06.013. 28709821 10.1016/j.jfma.2017.06.013

[17] Constantino J, Gruber C. Social Responsiveness Scale (SRS) Manual. Los Angeles, CA: Western Psychological Services; 2005.

[18] Courchesne V, Meilleur A-AS, Poulin-Lord M-P, Dawson M, Soulières I. Autistic children at risk of being underestimated: school-based pilot study of a strength-informed assessment. Mol Autism. 2015;6(1):12. 10.1186/s13229-015-0006-3. 25774281 10.1186/s13229-015-0006-3 PMC4359559

[19] Demetriou L, Kowalczyk OS, Tyson G, Bello T, Newbould RD, Wall MB. A comprehensive evaluation of increasing temporal resolution with multiband-accelerated protocols and effects on statistical outcome measures in fMRI. Neuroimage. 2018;176:404–16. 10.1016/j.neuroimage.2018.05.011. 29738911 10.1016/j.neuroimage.2018.05.011

[20] Dickie EW, Ameis SH, Shahab S, Calarco N, Smith DE, Miranda D, et al. Personalised intrinsic network topography mapping and functional connectivity deficits in Autism Spectrum Disorder. Biol Psychiatry. 2018;84(4):278–86. 10.1016/j.biopsych.2018.02.1174. 29703592 10.1016/j.biopsych.2018.02.1174 PMC6076333

[21] Diedrichsen J, Balsters JH, Flavell J, Cussans E, Ramnani N. A probabilistic MR atlas of the human cerebellum. Neuroimage. 2009;46(1):39–46. 10.1016/j.neuroimage.2009.01.045. 19457380 10.1016/j.neuroimage.2009.01.045

[22] Downing PE, Jiang Y, Shuman M, Kanwisher N. A cortical area selective for visual processing of the human body. Science. 2001;293(5539):2470–3. 10.1126/science.1063414. 11577239 10.1126/science.1063414

[23] Duan X, Shan X, Uddin LQ, Chen H. The future of disentangling the heterogeneity of autism with neuroimaging studies. Biol Psychiatry. 2025;97(5):428–38. 10.1016/j.biopsych.2024.08.008. 39181387 10.1016/j.biopsych.2024.08.008

[24] DuPre E, Salo T, Ahmed Z, Bandettini PA, Bottenhorn KL, Caballero-Gaudes C, et al. TE-dependent analysis of multi-echo fMRI with *tedana*. J Open Source Softw. 2021;6(66):3669. 10.21105/joss.03669.

[25] Endicott J, Spitzer RL. A diagnostic interview: the schedule for affective disorders and schizophrenia. Arch Gen Psychiatry. 1978;35(7):837–44. 10.1001/archpsyc.1978.01770310043002. 678037 10.1001/archpsyc.1978.01770310043002

[26] Fazal Z, Gomez DEP, Llera A, Marques JPRF, Beck T, Poser BA, et al. A comparison of multiband and multiband multiecho gradient-echo EPI for task fMRI at 3 T. Hum Brain Mapp. 2023;44(1):82–93. 10.1002/hbm.26081. 36196782 10.1002/hbm.26081 PMC9783458

[27] Federici A, Parma V, Vicovaro M, Radassao L, Casartelli L, Ronconi L. Anomalous perception of biological motion in autism: a conceptual review and meta-analysis. Sci Rep. 2020;10(1):1. 10.1038/s41598-020-61252-3. 32165647 10.1038/s41598-020-61252-3 PMC7067769

[28] Fidler DJ, Lanfranchi S. Executive function and intellectual disability: innovations, methods, and treatment. J Intellect Disabil Res. 2022;66(1–2):1–8. 10.1111/jir.12906. 34888975 10.1111/jir.12906 PMC8766896

[29] Foglia V, Siddiqui H, Khan Z, Liang S, Rutherford MD. Distinct biological motion perception in autism spectrum disorder: a meta-analysis. J Autism Dev Disord. 2022;52(11):4843–60. 10.1007/s10803-021-05352-7. 34783992 10.1007/s10803-021-05352-7 PMC9556430

[30] Freitag CM, Konrad C, Häberlen M, Kleser C, von Gontard A, Reith W, et al. Perception of biological motion in autism spectrum disorders. Neuropsychologia. 2008;46(5):1480–94. 10.1016/j.neuropsychologia.2007.12.025. 18262208 10.1016/j.neuropsychologia.2007.12.025

[31] Gallucci J, Pomarol-Clotet E, Voineskos A, Guerrero-Pedraza A, Alonso-Lana S, Vieta E, et al. Longer illness duration is associated with greater individual variability in functional brain activity in schizophrenia, but not bipolar disorder. Biol Psychiatry. 2023;93(9):S105. 10.1016/j.biopsych.2023.02.268. 10.1016/j.nicl.2022.103269 PMC9723315 36451371

[32] Gallucci J, Tan T, Schifani C, Dickie EW, Voineskos AN, Hawco C. Greater individual variability in functional brain activity during working memory performance in schizophrenia spectrum disorders (SSD). Schizophr Res. 2022;248:21–31. 10.1016/j.schres.2022.07.012. 35908378 10.1016/j.schres.2022.07.012

[33] Gau SS-F, Lin C-H, Hu F-C, Shang C-Y, Swanson JM, Liu Y-C, et al. Psychometric properties of the Chinese version of the Swanson, Nolan, and Pelham, version IV scale-teacher form. J Pediatr Psychol. 2009;34(8):850–61. 10.1093/jpepsy/jsn133. 19074488 10.1093/jpepsy/jsn133

[34] Gau SS-F, Liu L-T, Wu Y-Y, Chiu Y-N, Tsai W-C. Psychometric properties of the Chinese version of the Social Responsiveness Scale. Res Autism Spectr Disord. 2013;7(2):349–60. 10.1016/j.rasd.2012.10.004.

[35] Gilmore AW, Agron AM, González-Araya EI, Gotts SJ, Martin A. A comparison of single- and multi-echo processing of functional MRI data during overt autobiographical recall. Front Neurosci. 2022;16:854387. 10.3389/fnins.2022.854387. 35546886 10.3389/fnins.2022.854387 PMC9081814

[36] Gioia GA, Isquith PK, Guy SC, Kenworthy L. TEST REVIEW Behavior Rating Inventory of Executive Function. Child Neuropsychol. 2000;6(3):235–8. 10.1076/chin.6.3.235.3152. 11419452 10.1076/chin.6.3.235.3152

[37] Gollo LL, Roberts JA, Cropley VL, Di Biase MA, Pantelis C, Zalesky A, et al. Fragility and volatility of structural hubs in the human connectome. Nat Neurosci. 2018;21(8):1107–16. 10.1038/s41593-018-0188-z. 30038275 10.1038/s41593-018-0188-z

[38] Gotham K, Pickles A, Lord C. Standardizing ADOS scores for a measure of severity in autism spectrum disorders. J Autism Dev Disord. 2009;39(5):693–705. 10.1007/s10803-008-0674-3. 19082876 10.1007/s10803-008-0674-3 PMC2922918

[39] Grossman ED, Blake R. Brain areas active during visual perception of biological motion. Neuron. 2002;35(6):1167–75. 10.1016/S0896-6273(02)00897-8. 12354405 10.1016/s0896-6273(02)00897-8

[40] Hahamy A, Behrmann M, Malach R. The idiosyncratic brain: distortion of spontaneous connectivity patterns in autism spectrum disorder. Nat Neurosci. 2015;18(2):302–9. 10.1038/nn.3919. 25599222 10.1038/nn.3919

[41] Hasson U, Avidan G, Gelbard H, Vallines I, Harel M, Minshew N, et al. Shared and idiosyncratic cortical activation patterns in autism revealed under continuous real-life viewing conditions. Autism Res. 2009;2(4):220–31. 10.1002/aur.89. 19708061 10.1002/aur.89 PMC2775929

[42] Hawco C, Yoganathan L, Voineskos AN, Lyon R, Tan T, Daskalakis ZJ, et al. Greater individual variability in functional brain activity during working memory performance in young people with autism and executive function impairment. Neuroimage Clin. 2020;27:102260. 10.1016/j.nicl.2020.102260. 32388347 10.1016/j.nicl.2020.102260 PMC7218076

[43] Heberlein AS, Adolphs R, Tranel D, Damasio H. Cortical regions for judgments of emotions and personality traits from point-light walkers. J Cogn Neurosci. 2004;16(7):1143–58. 10.1162/0898929041920423. 15453970 10.1162/0898929041920423

[44] Herrington JD, Nymberg C, Schultz RT. Biological motion task performance predicts superior temporal sulcus activity. Brain Cogn. 2011;77(3):372–81. 10.1016/j.bandc.2011.09.001. 22024246 10.1016/j.bandc.2011.09.001

[45] Huang C-F, Lin Y-S, Chiu Y-N, Gau SS-F, Chen VC-H, Lin C-F, et al. Validation of the Chinese Version of the Autism Diagnostic Interview-Revised in Autism Spectrum Disorder. Neuropsychiatr Dis Treat. 2022;18:327. 10.2147/NDT.S345568. 35210779 10.2147/NDT.S345568 PMC8863335

[46] Hus V, Gotham K, Lord C. Standardizing ADOS domain scores: separating severity of social affect and restricted and repetitive behaviors. J Autism Dev Disord. 2014;44(10):2400–12. 10.1007/s10803-012-1719-1. 23143131 10.1007/s10803-012-1719-1 PMC3612387

[47] Jack A, Keifer CM, Pelphrey KA. Cerebellar contributions to biological motion perception in autism and typical development. Hum Brain Mapp. 2017;38(4):1914–32. 10.1002/hbm.23493. 28150911 10.1002/hbm.23493 PMC5342927

[48] Jack A, Pelphrey K. Annual research review: understudied populations within the autism spectrum - current trends and future directions in neuroimaging research. J Child Psychol Psychiatry Allied Discip. 2017;58(4):411–35. 10.1111/jcpp.12687. 10.1111/jcpp.12687 PMC5367938 28102566

[49] Johansson G. Visual perception of biological motion and a model for its analysis. Percept Psychophys. 1973;14(2):201–11. 10.3758/BF03212378.

[50] Kaiser MD, Hudac CM, Shultz S, Lee SM, Cheung C, Berken AM, et al. Neural signatures of autism. Proc Natl Acad Sci U S A. 2010;107(49):21223–8. 10.1073/pnas.1010412107. 21078973 10.1073/pnas.1010412107 PMC3000300

[51] Kaliukhovich DA, Manyakov NV, Bangerter A, Ness S, Skalkin A, Boice M, et al. Visual preference for biological motion in children and adults with autism spectrum disorder: an eye-tracking study. J Autism Dev Disord. 2021;51(7):2369–80. 10.1007/s10803-020-04707-w. 32951157 10.1007/s10803-020-04707-w PMC8189980

[52] Klin A, Lin DJ, Gorrindo P, Ramsay G, Jones W. Two-year-olds with autism orient to non-social contingencies rather than biological motion. Nature. 2009;459(7244):257–61. 10.1038/nature07868. 19329996 10.1038/nature07868 PMC2758571

[53] Knight EJ, Krakowski AI, Freedman EG, Butler JS, Molholm S, Foxe JJ. Attentional influences on neural processing of biological motion in typically developing children and those on the autism spectrum. Mol Autism. 2022;13(1):33. 10.1186/s13229-022-00512-7. 35850696 10.1186/s13229-022-00512-7 PMC9290301

[54] Kundu P, Brenowitz ND, Voon V, Worbe Y, Vértes PE, Inati SJ, et al. Integrated strategy for improving functional connectivity mapping using multiecho fMRI. Proc Natl Acad Sci U S A. 2013;110(40):16187–92. 10.1073/pnas.1301725110. 24038744 10.1073/pnas.1301725110 PMC3791700

[55] Lin HY, Lai MC. The Neuroradiology of Autism: Framing Neuroimaging Investigations of the Autistic Brain Based on the US NIMH Research Domain Criteria. In D. D. Eisenstat, D. Goldowitz, T. F. Oberlander, & J. Y. Yager (Eds.), Neurodevelopmental Pediatrics: Genetic and Environmental Influences (pp. 269–282). 2023. Springer International Publishing. 10.1007/978-3-031-20792-1_16.

[56] Lombardo MV, Lai M-C, Baron-Cohen S. Big data approaches to decomposing heterogeneity across the autism spectrum. Mol Psychiatry. 2019;24(10):1435. 10.1038/s41380-018-0321-0. 30617272 10.1038/s41380-018-0321-0 PMC6754748

[57] Lord C, Brugha TS, Charman T, Cusack J, Dumas G, Frazier T, et al. Autism spectrum disorder. Nat Rev Dis Primers. 2020;6(1):1–23. 10.1038/s41572-019-0138-4. 31949163 10.1038/s41572-019-0138-4 PMC8900942

[58] Lord C, Risi S, Lambrecht L, Cook EH, Leventhal BL, DiLavore PC, et al. The autism diagnostic observation schedule-generic: a standard measure of social and communication deficits associated with the spectrum of autism. J Autism Dev Disord. 2000;30(3):205–23. 11055457

[59] Lord C, Rutter M, Le Couteur A. Autism diagnostic interview-revised: a revised version of a diagnostic interview for caregivers of individuals with possible pervasive developmental disorders. J Autism Dev Disord. 1994;24(5):659–85. 10.1007/BF02172145. 7814313 10.1007/BF02172145

[60] Lu T. Adaptive behavior of the mentally retarded in Taiwan, ROC. Bull Spec Educ. 1993;9:107–44.

[61] McIntosh D, Miller L, Shyu V, Dunn W. Development and validation of the short sensory profile. Sensory Profile Manual. 1999;61:59–73.

[62] Mecca TP, Orsati FT, Macedo ECde. Non-verbal cognitive profile of young children with autism spectrum disorders. Psychology. 2014;5(11):1404–17. 10.4236/psych.2014.511151.

[63] Mishra P, Pandey CM, Singh U, Gupta A, Sahu C, Keshri A. Descriptive statistics and normality tests for statistical data. Ann Card Anaesth. 2019;22(1):67. 10.4103/aca.ACA_157_18. 30648682 10.4103/aca.ACA_157_18 PMC6350423

[64] Mottron L, Belleville S, Rouleau GA, Collignon O. Linking neocortical, cognitive, and genetic variability in autism with alterations of brain plasticity: the trigger-threshold-target model. Neurosci Biobehav Rev. 2014;47:735–52. 10.1016/j.neubiorev.2014.07.012. 25155242 10.1016/j.neubiorev.2014.07.012

[65] Mueller S, Wang D, Fox MD, Yeo BTT, Sepulcre J, Sabuncu MR, et al. Individual variability in functional connectivity architecture of the human brain. Neuron. 2013;77(3):586–95. 10.1016/j.neuron.2012.12.028. 23395382 10.1016/j.neuron.2012.12.028 PMC3746075

[66] Nunes AS, Peatfield N, Vakorin V, Doesburg SM. Idiosyncratic organization of cortical networks in autism spectrum disorder. Neuroimage. 2019;190:182–90. 10.1016/j.neuroimage.2018.01.022. 29355768 10.1016/j.neuroimage.2018.01.022

[67] Olsson MB, Holm A, Westerlund J, Hedvall ÅL, Gillberg C, Fernell E. Children with borderline intellectual functioning and autism spectrum disorder: Developmental trajectories from 4 to 11 years of age. Neuropsychiatr Dis Treat. 2017;13:2519. 10.2147/NDT.S143234. 29042781 10.2147/NDT.S143234 PMC5634384

[68] Ou W, Zeng W, Gao W, He J, Meng Y, Fang X, et al. Movie events detecting reveals inter-subject synchrony difference of functional brain activity in autism spectrum disorder. Front Comput Neurosci. 2022;16:877204. 10.3389/fncom.2022.877204. 35591883 10.3389/fncom.2022.877204 PMC9110681

[69] Poulin-Lord M-P, Barbeau EB, Soulières I, Monchi O, Doyon J, Benali H, et al. Increased topographical variability of task-related activation in perceptive and motor associative regions in adult autistics. Neuroimage Clin. 2014;4:444–53. 10.1016/j.nicl.2014.02.008. 25101235 10.1016/j.nicl.2014.02.008 PMC4116759

[70] Prakash A, Banerjee M. Genomic selection signatures in autism spectrum disorder identifies cognitive genomic tradeoff and its relevance in paradoxical phenotypes of deficits versus potentialities. Sci Rep. 2021;11(1):10245. 10.1038/s41598-021-89798-w. 33986442 10.1038/s41598-021-89798-w PMC8119484

[71] Roid GH, Miller LJ. Leiter-R : Leiter international performance scale-revised. Stoelting. WorldCat. 2002.

[72] Rutherford MD. Evidence for Specialized Perception of Animate Motion. In M. D. Rutherford & V. A. Kuhlmeier (Eds.), *Social Perception* (pp. 115–138). The MIT Press. 2013. https://academic.oup.com/mit-press-scholarship-online/book/14726/chapter/168912994.

[73] Salmi J, Roine U, Glerean E, Lahnakoski J, von Nieminen- Wendt T, Tani P, et al. The brains of high functioning autistic individuals do not synchronize with those of others. Neuroimage Clin. 2013;3:489–97. 10.1016/j.nicl.2013.10.011. 24273731 10.1016/j.nicl.2013.10.011 PMC3830058

[74] Schaefer A, Kong R, Gordon EM, Laumann TO, Zuo X-N, Holmes AJ, et al. Local-global parcellation of the human cerebral cortex from intrinsic functional connectivity MRI. Cerebral Cortex (New York, NY: 1991). 2018;28(9):3095–114. 10.1093/cercor/bhx179. 10.1093/cercor/bhx179 PMC6095216 28981612

[75] Scherf KS, Luna B, Minshew N, Behrmann M. Location, location, location: alterations in the functional topography of face- but not object- or place-related cortex in adolescents with autism. Front Hum Neurosci. 2010;4:26. 10.3389/fnhum.2010.00026. 20631857 10.3389/fnhum.2010.00026 PMC2904054

[76] Schifani C, Hawco C, Daskalakis ZJ, Rajji TK, Mulsant BH, Tan V, et al. Repetitive Transcranial Magnetic Stimulation (rTMS) Treatment Reduces Variability in Brain Function in Schizophrenia: Data From a Double-Blind, Randomized. Sham-Controlled Trial Schizophrenia Bulletin. 2025;51(3):818–28. 10.1093/schbul/sbae166. 39373168 10.1093/schbul/sbae166 PMC12061648

[77] Schultz J, Bülthoff HH. Perceiving animacy purely from visual motion cues involves intraparietal sulcus. Neuroimage. 2019;197:120–32. 10.1016/j.neuroimage.2019.04.058. 31028922 10.1016/j.neuroimage.2019.04.058

[78] Secara MT, Oliver LD, Gallucci J, Dickie EW, Foussias G, Gold J, et al. Heterogeneity in functional connectivity: dimensional predictors of individual variability during rest and task fMRI in psychosis. Prog Neuropsychopharmacol Biol Psychiatry. 2024;132:110991. 10.1016/j.pnpbp.2024.110991. 38484928 10.1016/j.pnpbp.2024.110991 PMC11034852

[79] Smith SM, Nichols TE. Threshold-free cluster enhancement: addressing problems of smoothing, threshold dependence and localisation in cluster inference. Neuroimage. 2009;44(1):83–98. 10.1016/j.neuroimage.2008.03.061. 18501637 10.1016/j.neuroimage.2008.03.061

[80] Sokolov AA, Erb M, Gharabaghi A, Grodd W, Tatagiba MS, Pavlova MA. Biological motion processing: the left cerebellum communicates with the right superior temporal sulcus. Neuroimage. 2012;59(3):2824–30. 10.1016/j.neuroimage.2011.08.039. 22019860 10.1016/j.neuroimage.2011.08.039

[81] Souto D, Sudkamp J, Nacilla K, Bocian M. Tuning in to a hip-hop beat: pursuit eye movements reveal processing of biological motion. Hum Mov Sci. 2023;91:103126. 10.1016/j.humov.2023.103126. 37517315 10.1016/j.humov.2023.103126

[82] Sparrow SS, Cicchetti DV. The Vineland Adaptive Behavior Scales. In Major psychological assessment instruments. Allyn & Bacon. 1989;2:199-231.

[83] Sridhar A, Joanne Jao Keehn R, Wilkinson M, Gao Y, Olson M, Mash LE, et al. Increased heterogeneity and task-related reconfiguration of functional connectivity during a lexicosemantic task in autism. Neuroimage Clin. 2024;44:103694. 10.1016/j.nicl.2024.103694. 39509989 10.1016/j.nicl.2024.103694 PMC11574795

[84] Steel A, Garcia BD, Silson EH, Robertson CE. Evaluating the efficacy of multi-echo ICA denoising on model-based fMRI. Neuroimage. 2022;264:119723. 10.1016/j.neuroimage.2022.119723. 36328274 10.1016/j.neuroimage.2022.119723

[85] Swanson JM. Appendix 5—Chapter 2 The SNAP-IV Teacher and Parent Rating Scale. In A. H. Fine & R. A. Kotkin (Eds.), Therapist’s Guide to Learning and Attention Disorders. 2003:487-500 Academic Press. https://www.sciencedirect.com/science/article/pii/B9780122564307500223.

[86] Tan V, Downar J, Nestor S, Vila-Rodriguez F, Daskalakis ZJ, Blumberger DM, et al. Effects of repetitive transcranial magnetic stimulation on individual variability of resting-state functional connectivity in major depressive disorder. J Psychiatr Neurosci: JPN. 2024;49(3):E172–81. 10.1503/jpn.230135. 10.1503/jpn.230135 PMC11090631 38729664

[87] Thompson J, Parasuraman R. Attention, biological motion, and action recognition. NeuroImage, Neuroergonomics: The Human Brain in Action and at Work. 2012;59(1):4–13. 10.1016/j.neuroimage.2011.05.044. 10.1016/j.neuroimage.2011.05.044 21640836

[88] Thurm A, Farmer C, Salzman E, Lord C, Bishop S. State of the field: differentiating intellectual disability from autism spectrum disorder. Front Psychiatry. 2019;10:526. 10.3389/fpsyt.2019.00526. 31417436 10.3389/fpsyt.2019.00526 PMC6683759

[89] Tian J, Yang F, Wang Y, Wang L, Wang N, Jiang Y, et al. A typical biological motion perception in children with attention deficit hyperactivity disorder: local motion and global configuration processing. Elife. 2024;12:RP90313. 10.7554/eLife.90313.4. 38954462 10.7554/eLife.90313 PMC11219041

[90] Tian Y, Margulies DS, Breakspear M, Zalesky A. Topographic organization of the human subcortex unveiled with functional connectivity gradients. Nat Neurosci. 2020;23(11):1421–32. 10.1038/s41593-020-00711-6. 32989295 10.1038/s41593-020-00711-6

[91] Todorova GK, Hatton REM, Pollick FE. Biological motion perception in autism spectrum disorder: a meta-analysis. Mol Autism. 2019;10:49. 10.1186/s13229-019-0299-8. 31890147 10.1186/s13229-019-0299-8 PMC6921539

[92] Troje NF, Westhoff C. The inversion effect in biological motion perception: evidence for a “life detector”? Curr Biol. 2006;16(8):821–4. 10.1016/j.cub.2006.03.022. 16631591 10.1016/j.cub.2006.03.022

[93] Tung Y-H, Lin H-Y, Chen C-L, Shang C-Y, Yang L-Y, Hsu Y-C, et al. Whole brain white matter tract deviation and idiosyncrasy from normative development in autism and ADHD and unaffected siblings link with dimensions of psychopathology and cognition. Am J Psychiatry. 2021;178(8):730–43. 10.1176/appi.ajp.2020.20070999. 33726525 10.1176/appi.ajp.2020.20070999

[94] Vaina LM, Solomon J, Chowdhury S, Sinha P, Belliveau JW. Functional neuroanatomy of biological motion perception in humans. Proc Natl Acad Sci U S A. 2001;98(20):11656–61. 10.1073/pnas.191374198. 11553776 10.1073/pnas.191374198 PMC58785

[95] Wechsler D. WISC-IV: Weschler Intelligence Scale for Children- fourth edition. Pearson. 2003.

[96] Wechsler D. WAIS-IV : Wechsler adult intelligence scale (4th ed). Pearson. WorldCat. 2008.

[97] Wheaton KJ, Pipingas A, Silberstein RB, Puce A. Human neural responses elicited to observing the actions of others. Vis Neurosci. 2001;18(3):401–6. 10.1017/S0952523801183069. 11497416 10.1017/s0952523801183069

[98] Yan C-G, Craddock RC, Zuo X-N, Zang Y-F, Milham MP. Standardizing the intrinsic brain: towards robust measurement of inter-individual variation in 1000 functional connectomes. Neuroimage. 2013;80:246–62. 10.1016/j.neuroimage.2013.04.081. 23631983 10.1016/j.neuroimage.2013.04.081 PMC4074397

[99] Yang D, Svoboda AM, George TG, Mansfield PK, Wheelock MD, Schroeder ML, et al. Mapping neural correlates of biological motion perception in autistic children using high-density diffuse optical tomography. Mol Autism. 2024;15(1):35. 10.1186/s13229-024-00614-4. 39175054 10.1186/s13229-024-00614-4 PMC11342641

[100] Yang YJD, Sukhodolsky DG, Lei J, Dayan E, Pelphrey KA, Ventola P. Distinct neural bases of disruptive behavior and autism symptom severity in boys with autism spectrum disorder. J Neurodev Disord. 2017;9(1):1. 10.1186/s11689-017-9183-z. 28115995 10.1186/s11689-017-9183-z PMC5240249

[101] Yeh C-H, Tseng R-Y, Ni H-C, Cocchi L, Chang J-C, Hsu M-Y, et al. White matter microstructural and morphometric alterations in autism: implications for intellectual capabilities. Mol Autism. 2022;13(1):21. 10.1186/s13229-022-00499-1. 35585645 10.1186/s13229-022-00499-1 PMC9118608

