# Supplementary material for: Brain idiosyncrasy during biological-motion perception is amplified in autistic individuals with intellectual impairment
Source: J Neurodev Disord. 2026 Mar 25;18:31. doi: 10.1186/s11689-026-09683-3 (PMC13217712; doi:10.1186/s11689-026-09683-3)
Supplement: Supplementary file 1 — Supplementary Material 1. [file 11689_2026_9683_MOESM1_ESM.docx]

**Supporting Information**

**Brain idiosyncrasy during biological-motion perception is amplified in autistic individuals with intellectual impairment**

**Supplementary Methods**

**Multi-echo denoising**

An initial mask was generated from the first echo using nilearn’s compute_epi_mask function. An adaptive mask was then generated, in which each voxel’s value reflects the number of echoes with “good” data. A monoexponential model was fitted to the data at each voxel using log-linear regression to estimate T2* and S0 maps. For each voxel, the value from the adaptive mask was used to determine which echoes would be used to estimate T2* and S0. Multi-echo data were optimally combined using the ‘t2s’ combination method. Principal component analysis followed by the Kundu component selection decision tree was applied to the optimally combined data for dimensionality reduction. ICA was then used to decompose the dimensionally reduced dataset. A series of TE-dependence metrics were calculated for each ICA component, including Kappa, Rho, and variance explained. Next, automatic component classification was performed to identify BOLD (TE-dependent), non-BOLD/noise (TE-independent), and uncertain (low-variance) components using the Kundu decision tree (v3.2) (Kundu et al., 2013), whereby denoising was done by removing non-BOLD components from the optimally combined data. T1c global signal regression was then applied to the data to remove spatially diffuse noise. ME-ICA's robust denoising allowed us to skip spatial smoothing, aiming to preserve interindividual spatial variability (Kundu et al., 2017; Mueller et al., 2013).

**Supplementary References**

Kundu, P., Brenowitz, N. D., Voon, V., Worbe, Y., Vértes, P. E., Inati, S. J., Saad, Z. S., Bandettini, P. A., & Bullmore, E. T. (2013). Integrated strategy for improving functional connectivity mapping using multiecho fMRI. *Proceedings of the National Academy of Sciences of the United States of America*, *110*(40), 16187–16192. https://doi.org/10.1073/pnas.1301725110

Kundu, P., Voon, V., Balchandani, P., Lombardo, M. V., Poser, B. A., & Bandettini, P. A. (2017). Multi-echo fMRI: A review of applications in fMRI denoising and analysis of BOLD signals. *NeuroImage, Cleaning up the fMRI Time Series: Mitigating Noise with Advanced Acquisition and Correction Strategies*, *154*, 59–80. https://doi.org/10.1016/j.neuroimage.2017.03.033

Mueller, S., Wang, D., Fox, M. D., Yeo, B. T. T., Sepulcre, J., Sabuncu, M. R., Shafee, R., Lu, J., & Liu, H. (2013). Individual variability in functional connectivity architecture of the human brain. *Neuron*, *77*(3), 586–595. https://doi.org/10.1016/j.neuron.2012.12.028

Wood, S. N. (2011). Fast Stable Restricted Maximum Likelihood and Marginal Likelihood Estimation of Semiparametric Generalized Linear Models. *Journal of the Royal Statistical Society Series B: Statistical Methodology*, *73*(1), 3–36. https://doi.org/10.1111/j.1467-9868.2010.00749.x

**Table S1. Co-occurring neuropsychiatric conditions and medication use for ASC-IA & ASC-II**

|  | ASC-IA (n= 28) | ASC-II (n= 19) |
| --- | --- | --- |
| **Comorbidity** | | |
| No co-occurring neuropsychiatric conditions | 8 | 3 |
| Co-occurring ADHD | 11 | 8 |
| Co-occurring Anxiety disorder | 7 (5 social anxiety disorder; 2 specific phobia) | 6 (3 specific phobia; 2 social anxiety disorder; 1 selective mutism) |
| Co-occurring tic disorder | 1 | 1 |
| Co-occurring OCD | 1 | 1 |
| Co-occurring learning disorder | 1 (writing/reading disorder) | N/A |
| Co-occurring depressive disorder | 1 (1 history of depression) | N/A |
| Other co-occurring conditions | 1 ODD | 1 epilepsy (well controlled) |
|  |  |  |
| **Medications** | | |
| Methylphenidate | 9 | 2 |
| Antidepressant | 2 (1 sertraline; 1 fluoxetine) | 1 (sertraline) |
| Valproic acid (mood stabilizer) | N/A | 1 |
| Antipsychotic | N/A | N/A |

Acronyms – ADHD: Attention Deficit/Hyperactivity Disorder; MDD: Major Depressive Disorder; OCD: Obsessive Compulsive Disorder; ODD: Oppositional Defiant Disorder.

**Table S2. Tests of statistical significance for brain-behaviour correlations**

|  | Behaviour | | | Age | | | Sex | | | Mean FD | | | Whole Model |
| --- | --- | --- | --- | --- | --- | --- | --- | --- | --- | --- | --- | --- | --- |
|  | F/T | p | q | F/T | p | q | F/T | p | q | F/T | p | q | Adjusted R^2^ |
| NVIQ | 8.11^b^ | 6.64*10^-4^ | 0.0052 | -2.14^a^ | 0.036 | 0.23 | 0.57^a^ | 0.57 | 0.64 | 1.47^b^ | 0.23 | 0.26 | 0.25 |
| RBS-R | 5.78^b^ | 0.019 | 0.030 | -1.93^a^ | 0.057 | 0.23 | 1.43^a^ | 0.16 | 0.60 | 1.74^b^ | 0.19 | 0.25 | 0.18 |
| SRS Total | 4.31^b^ | 0.041 | 0.047 | -1.74^a^ | 0.086 | 0.23 | 1.06^a^ | 0.30 | 0.60 | 2.90^b^ | 0.093 | 0.25 | 0.17 |
| SSP Total | 4.84^b^ | 0.031 | 0.041 | -1.47^a^ | 0.15 | 0.23 | 0.96^a^ | 0.34 | 0.60 | 1.95^b^ | 0.17 | 0.25 | 0.14 |
| ADOS-2 CSS | 1.62^a^ | 0.11 | 0.11 | -1.04^a^ | 0.30 | 0.30 | 0.47^a^ | 0.64 | 0.64 | 1.33^b^ | 0.26 | 0.26 | 0.087 |
| BRIEF-GEC | 2.40^a^ | 0.019 | 0.030 | -1.41^a^ | 0.16 | 0.23 | 0.90^a^ | 0.37 | 0.60 | 2.57^b^ | 0.11 | 0.25 | 0.18 |
| SNAP-IV Total | 3.35^a^ | 0.0013 | 0.0052 | -1.38^a^ | 0.17 | 0.23 | 0.70^a^ | 0.48 | 0.64 | 2.56^b^ | 0.11 | 0.25 | 0.24 |
| VABS-ABC | -2.48^a^ | 0.015 | 0.030 | -1.16^a^ | 0.25 | 0.29 | 1.29^a^ | 0.20 | 0.60 | 2.40^b^ | 0.13 | 0.25 | 0.19 |

The mgcv package in RStudio (Wood, 2011) was used to create generalized additive models. ^a^T-statistics were reported for terms treated as linear regressors. ^b^F-statistics were reported for terms treated as nonlinear regressors.

Acronyms – SNAP-IV: Swanson, Nolan, and Pelham ADHD Rating Scale; SRS: Social Responsiveness Scales; RBS-R: Repetitive Behavior Scale–Revised; BRIEF-GEC: Behavior Rating Inventory of Executive Function - Global Executive Composite; VABS-ABC: Vineland Adaptive Behavior Scales - Adaptive Behavior Composite; SSP: Short Sensory Profile; ADOS-2 CSS: Autism Diagnostic Observation Schedule-2 Calibrated Severity Score; NVIQ: Nonverbal Full-Scale Intelligence Quotient (Leiter-R)

**Table S3. Tests of statistical significance for group differences in idiosyncrasy in male participants only**

| TDC vs ASC-IA vs ASC-II |  |  |  | |  | |  | |  | |
| --- | --- | --- | --- | --- | --- | --- | --- | --- | --- | --- |
|  | df | SS | | MS | | F | | p | | η_p_^2^ |
| Group | 2 | 0.01317 | | 0.006587 | | 8.583 | | 0.00050 | | 0.1928 |
| Age | 1 | 0.006636 | | 0.006356 | | 8.282 | | 0.0054 | | 0.07208 |
| Mean FD | 1 | 0.00154 | | 0.001541 | | 2.008 | | 0.16 | | 0.0304 |
| Residuals | 64 | 0.04912 | | 0.000767 | |  | |  | |  |
|  |  |  | |  | |  | |  | |  |
| TDC vs ASC-Whole |  |  | |  | |  | |  | |  |
|  | df | SS | | MS | | F | | p | | η_p_^2^ |
| Group | 1 | 0.00620 | | 0.006204 | | 7.044 | | 0.010 | | 0.05916 |
| Age | 1 | 0.00477 | | 0.004774 | | 5.420 | | 0.023 | | 0.04098 |
| Mean FD | 1 | 0.00196 | | 0.001965 | | 2.231 | | 0.14 | | 0.03318 |
| Residuals | 65 | 0.05725 | | 0.000881 | |  | |  | |  |
|  |  |  | |  | |  | |  | |  |
| Post-hoc tests |  |  | |  | |  | |  | |  |
|  | Difference (unadjusted) | d | | p | | p_FWE_ | |  | |  |
| TDC-ASC-IA | -0.008755 | 0.3031 | | 0.27 | | 0.78 | |  | |  |
| TDC-ASC-II | -0.03417 | 1.1696 | | 0.00022 | | 0.00066 | |  | |  |
| ASC-II-ASC-IA | 0.02541 | 0.4427 | | 0.0057 | | 0.017 | |  | |  |
| TDC-ASC-Whole | -0.01972 | 0.6384 | | 0.010 | | - | |  | |  |

Post-hoc testing for TDC vs ASC-IA vs ASC-II was performed using pairwise ANCOVAs with Bonferroni correction. TDC vs ASC-Whole was tested separately.

**Table S4. Tests of statistical significance for brain-behaviour correlations in the male-only subsample**

|  | Behaviour | | | | Age | | | Mean FD | | | | | Whole Model |
| --- | --- | --- | --- | --- | --- | --- | --- | --- | --- | --- | --- | --- | --- |
|  | F/T | p | q | F/T | | p | q | | F/T | p | q | Adjusted R^2^ | |
| NVIQ | 6.88^b^ | 0.0019 | 0.0077 | -2.27^a^ | | 0.026 | 0.21 | | 1.45^b^ | 0.23 | 0.23 | 0.26 | |
| RBS-R | 5.63^b^ | 0.021 | 0.030 | -1.91^a^ | | 0.060 | 0.21 | | 1.69^b^ | 0.20 | 0.23 | 0.18 | |
| SRS Total | 5.44^b^ | 0.023 | 0.030 | -1.64^a^ | | 0.10 | 0.21 | | 2.99^b^ | 0.089 | 0.23 | 0.18 | |
| SSP Total | 5.18^b^ | 0.026 | 0.030 | -1.64^a^ | | 0.11 | 0.21 | | 1.91^b^ | 0.17 | 0.23 | 0.16 | |
| ADOS-2 CSS | 1.42^a^ | 0.16 | 0.16 | -1.02^a^ | | 0.32 | 0.32 | | 1.72^b^ | 0.20 | 0.23 | 0.082 | |
| BRIEF-GEC | 2.70^a^ | 0.0090 | 0.018 | -1.24^a^ | | 0.22 | 0.30 | | 2.76^b^ | 0.10 | 0.23 | 0.20 | |
| SNAP-IV Total | 3.51^a^ | 0.00086 | 0.0069 | -1.25^a^ | | 0.22 | 0.30 | | 2.89^b^ | 0.095 | 0.23 | 0.26 | |
| VABS-ABC | -2.70^a^ | 0.0089 | 0.018 | -1.06^a^ | | 0.29 | 0.32 | | 2.56^b^ | 0.11 | 0.23 | 0.18 | |

The mgcv package in RStudio (Wood, 2011) was used to create generalized additive models. ^a^T-statistics were reported for terms treated as linear regressors. ^b^F-statistics were reported for terms treated as nonlinear regressors.

Acronyms – SNAP-IV: Swanson, Nolan, and Pelham ADHD Rating Scale; SRS: Social Responsiveness Scales; RBS-R: Repetitive Behavior Scale–Revised; BRIEF-GEC: Behavior Rating Inventory of Executive Function - Global Executive Composite; VABS-ABC: Vineland Adaptive Behavior Scales - Adaptive Behavior Composite; SSP: Short Sensory Profile; ADOS-2 CSS: Autism Diagnostic Observation Schedule-2 Calibrated Severity Score; NVIQ: Nonverbal Full-Scale Intelligence Quotient (Leiter-R)


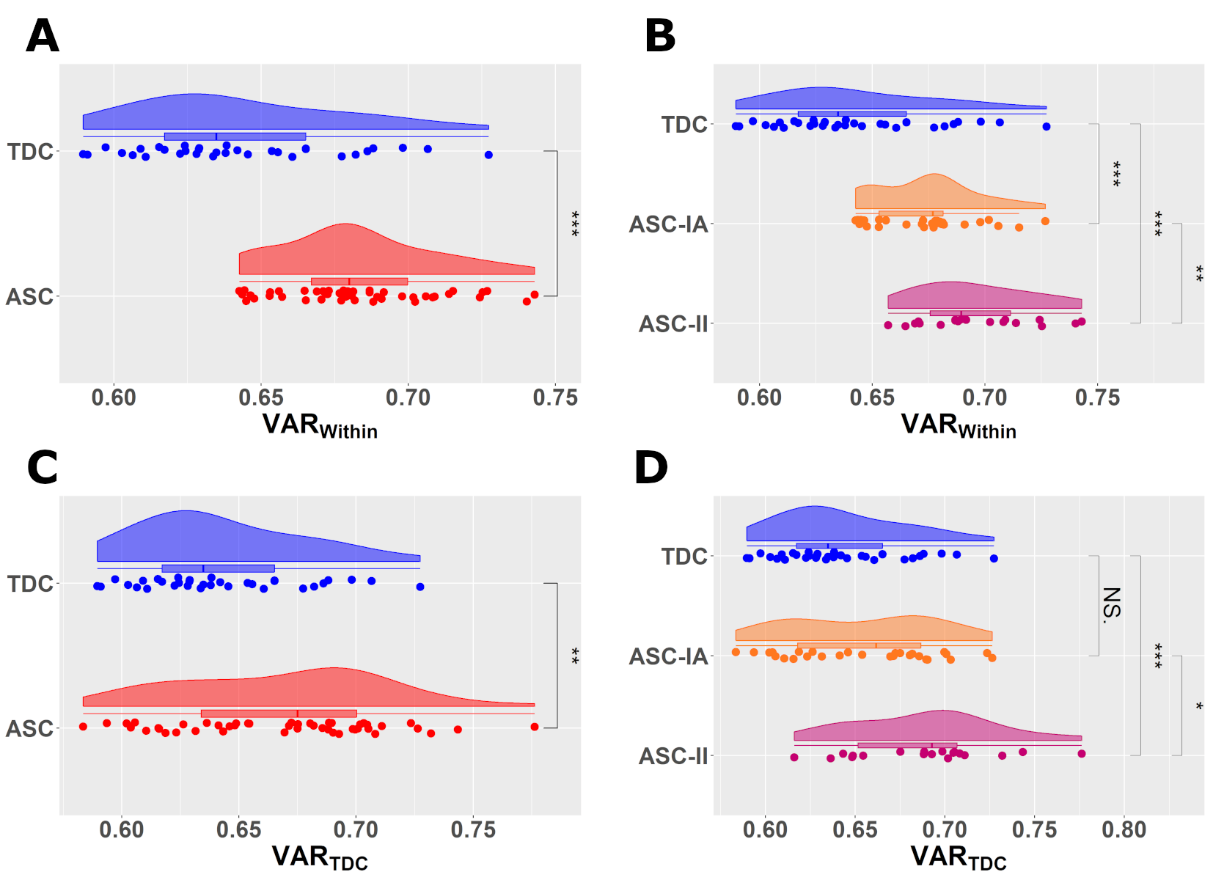


**Figure S1. Variability_Within_ and Variability_TDC_ across diagnostic groups.** Raincloud plots illustrate the distribution of “within-group variability” (Variability_Within_; compared to others in the same diagnostic group) and TDC reference variability (Variability_TDC_: compared to participants in TDC group) between participants. ANCOVA (controlling for age, sex, and mean FD) was used to assess group effects on variability. ASC-Whole (n=47) exhibited greater Variability_Within_ (A; ***p<0.001) and Variability_TDC_ (C; **p<0.01) than TDC (n=33). Significant group effects were also observed when stratifying the ASC group by IQ into ASC-IA and ASC-II (B, D). Post-hoc pairwise ANCOVA tests with Bonferroni correction showed that ASC-IA and ASC-II exhibited greater Variability_Within_ than TDC (B; ASC-IA vs. TDC: ***p_FWE_<0.001; ASC-II vs TDC: ***p_FWE_<0.001); ASC-II had greater Variability_Within_ than ASC-IA (B; **p_FWE_<0.01). When considering Variability_TDC_ (D), ASC-II was also more idiosyncratic than ASC-IA (*p_FWE_<0.05) and TDC (***p_FWE_<0.001) but ASC-IA did not significantly differ from TDC (p_FWE_>0.05). Individual data points are overlaid.


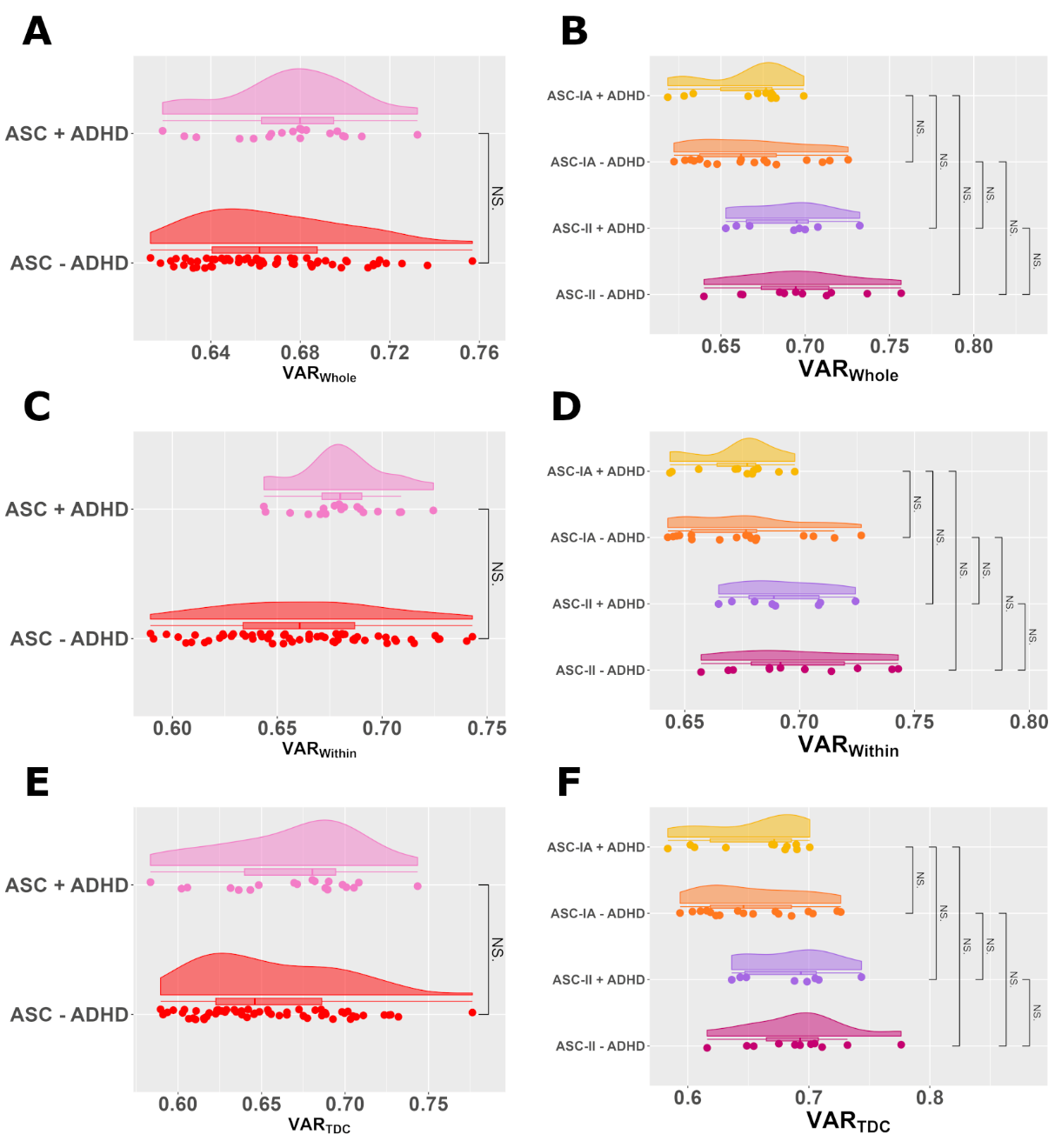


**Figure S2. Variability_Whole_, Variability_Within_, and Variability_TDC_ in participants with and without ADHD.** Raincloud plots illustrate the distribution of “whole-group variability (Variability_Whole_; compared to all other participants), “within-group variability” [Variability_Within_; compared to others in the same diagnostic group (i.e. ASC-IA or ASC-II)], and TDC reference variability (Variability_TDC_: compared to participants in TDC group) between autistic participants with and without ADHD (no TDC had co-occurring ADHD). ANCOVA (controlling for age, sex, and mean FD) was used to assess group effects on variability. There was no significant difference in Variability_Whole_ (A, F = 0.078, p = 0.78, η_p_^2^ = 3.2*10^-4^), Variability_Within_ (C, F = 0.23, p = 0.64, η_p_^2^ =2.2 * 10^-4^), or Variability_TDC_ (E, F = 0.010 , p = 0.92, η_p_^2^ =1.9 * 10^-3^) between autistic participants with (ASC + ADHD, n = 19) and without (ASC - ADHD, n = 28) ADHD. When stratifying each group by intellectual capacity [ASC-IA + ADHD (n = 11) vs ASC-IA - ADHD (n = 17) vs ASC-II + ADHD (n = 8) vs ASC-II - ADHD (n = 11)], the initial ANCOVA analyses showed a significant effect of group on Variability_Whole_ (B, F = 3.17, p = 0.035, η_p_^2^ = 0.18), Variability_Within_ (D, F = 3.52, p = 0.024, η_p_^2^ = 0.20), but not Variability_TDC_ (F, F = 2.61, p = 0.065, η_p_^2^ = 0.16). Post-hoc pairwise ANCOVA analyses were conducted, but no pairwise differences between groups remained significant following Bonferroni correction (p_FWE_ > 0.05). Individual data points are overlaid.

**
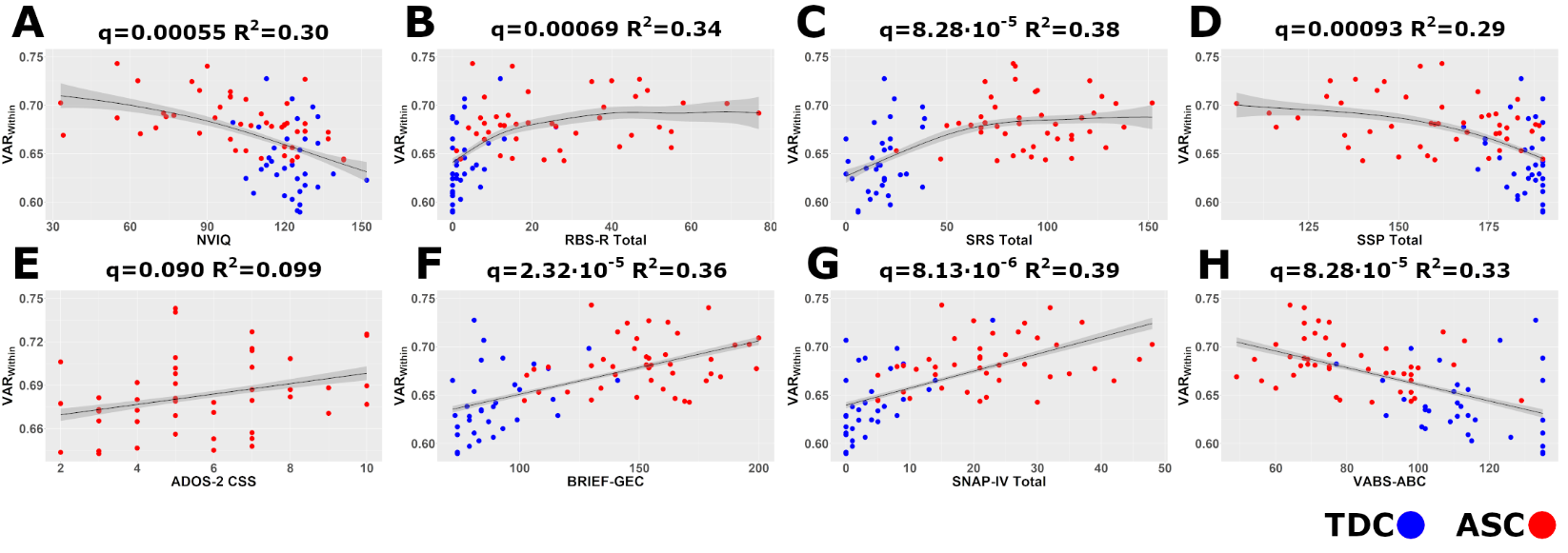
**

**Figure S3. Transdiagnostic associations between within-group neural idiosyncrasy (Variability_Within_) and cognitive/behavioural measures.** Scatterplots depict relationships across all participants, controlling for age, sex, and mean FD. Significant linear associations (FDR q<0.05) were found for BRIEF-GEC (F), SNAP-IV Total (G), and VABS-ABC (H). Significant non-linear associations (GAMs, FDR q<0.05) were found for NVIQ (A), RBS-R Total (B), SRS Total (C), and SSP Total (D) (lines show model fits). ADOS-2 CSS (E) was not significantly associated. Shaded areas represent 95% confidence intervals. Notably, higher SSP scores represent fewer sensory symptoms.

**
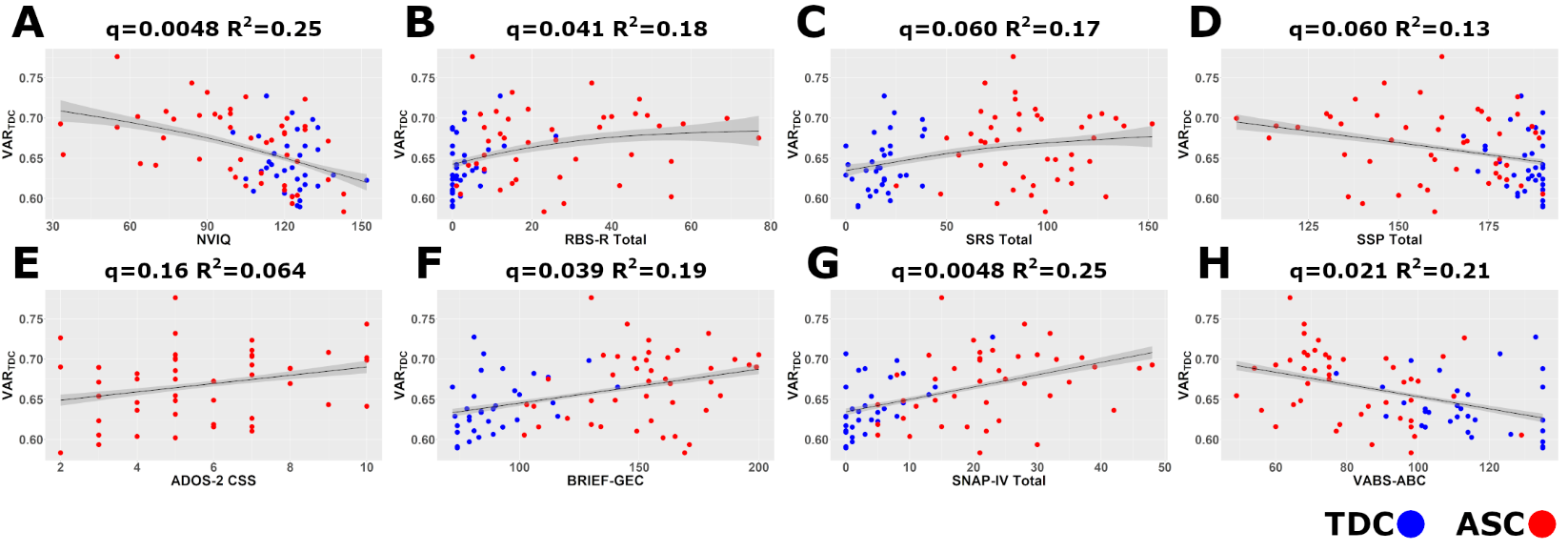
**

**Figure S4. Transdiagnostic associations between TDC-reference neural idiosyncrasy (Variability_TDC_) and cognitive/behavioural measures.** Scatterplots depict relationships across all participants, controlling for age, sex, and mean FD. Significant linear associations (FDR q<0.05) were found for BRIEF-GEC (F), SNAP-IV Total (G), and VABS-ABC (H). Significant non-linear associations (GAMs, FDR q<0.05) were found for NVIQ (A), RBS-R Total (B), and SSP Total (D) (lines show model fits). SRS Total (C) and ADOS-2 CSS (E) were not significantly associated. Shaded areas represent 95% confidence intervals. Notably, higher SSP scores represent fewer sensory symptoms.

**
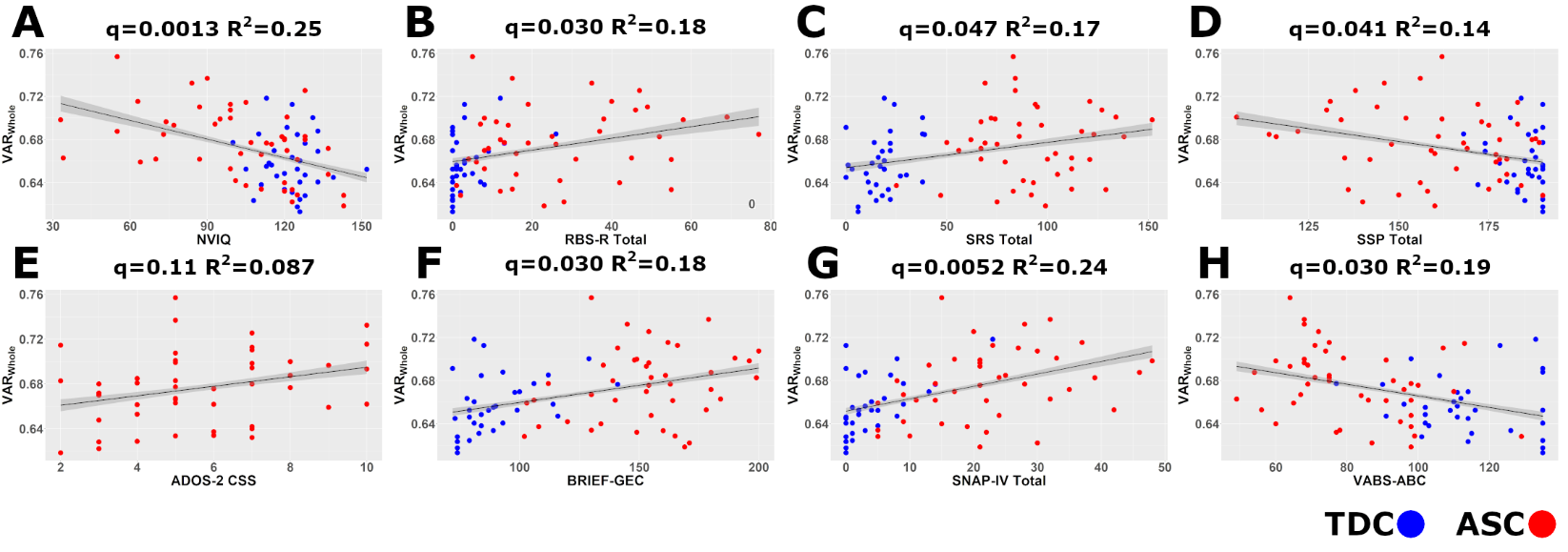
**

**Figure S5. Transdiagnostic linear correlations between whole-sample neural idiosyncrasy (Variability_Whole_) and cognitive/behavioural measures.** Scatterplots depict relationships across all participants, controlling for age, sex, and mean FD. Mean FD was treated as a smooth term in the GAMs since its distribution was not Gaussian. Significant linear associations (FDR q<0.05) were found for NVIQ (A), RBS-R Total (B), SRS Total (C), and SSP Total (D), BRIEF-GEC (F), SNAP-IV Total (G), and VABS-ABC (H). ADOS-2 CSS (E) was not significantly associated. Shaded areas represent 95% confidence intervals. Notably, higher SSP scores represent fewer sensory symptoms.

**
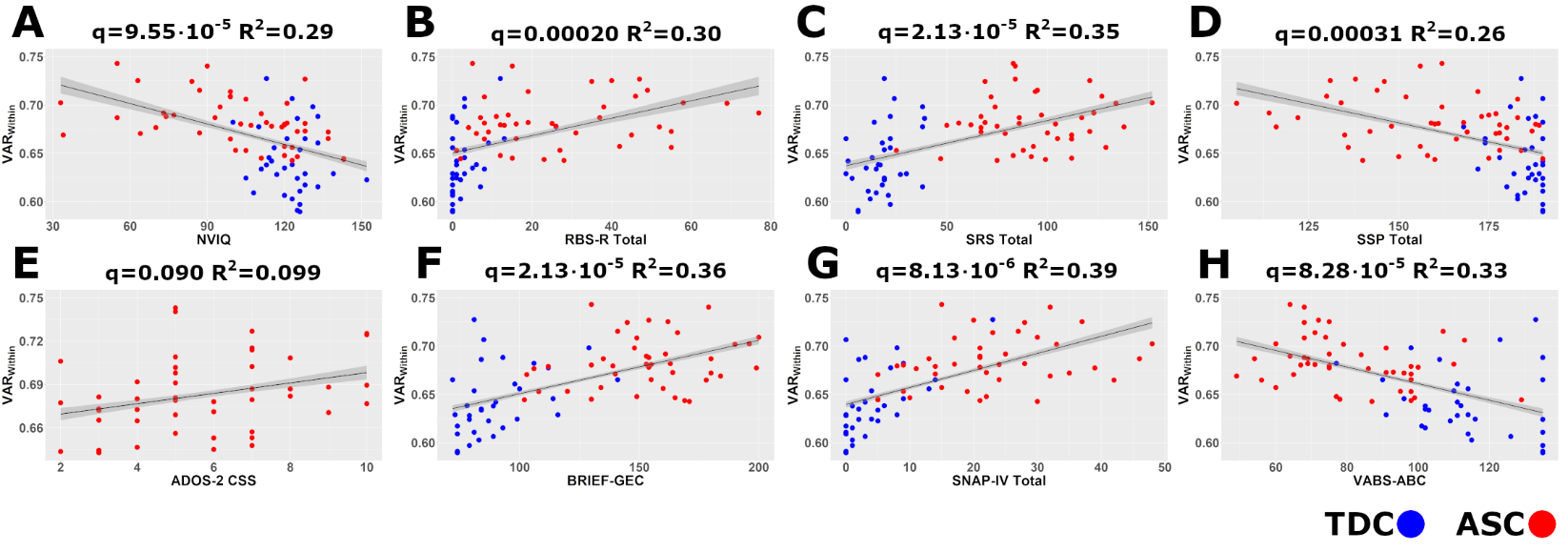
**

**Figure S6. Transdiagnostic linear correlations between within-group neural idiosyncrasy (Variability_Within_) and cognitive/behavioural measures.** Scatterplots depict relationships across all participants, controlling for age, sex, and mean FD. Mean FD was treated as a smooth term in the GAMs since its distribution was not Gaussian. Significant linear associations (FDR q<0.05) were found for NVIQ (A), RBS-R Total (B), SRS Total (C), and SSP Total (D), BRIEF-GEC (F), SNAP-IV Total (G), and VABS-ABC (H). ADOS-2 CSS (E) was not significantly associated. Shaded areas represent 95% confidence intervals. Notably, higher SSP scores represent fewer sensory symptoms.

**
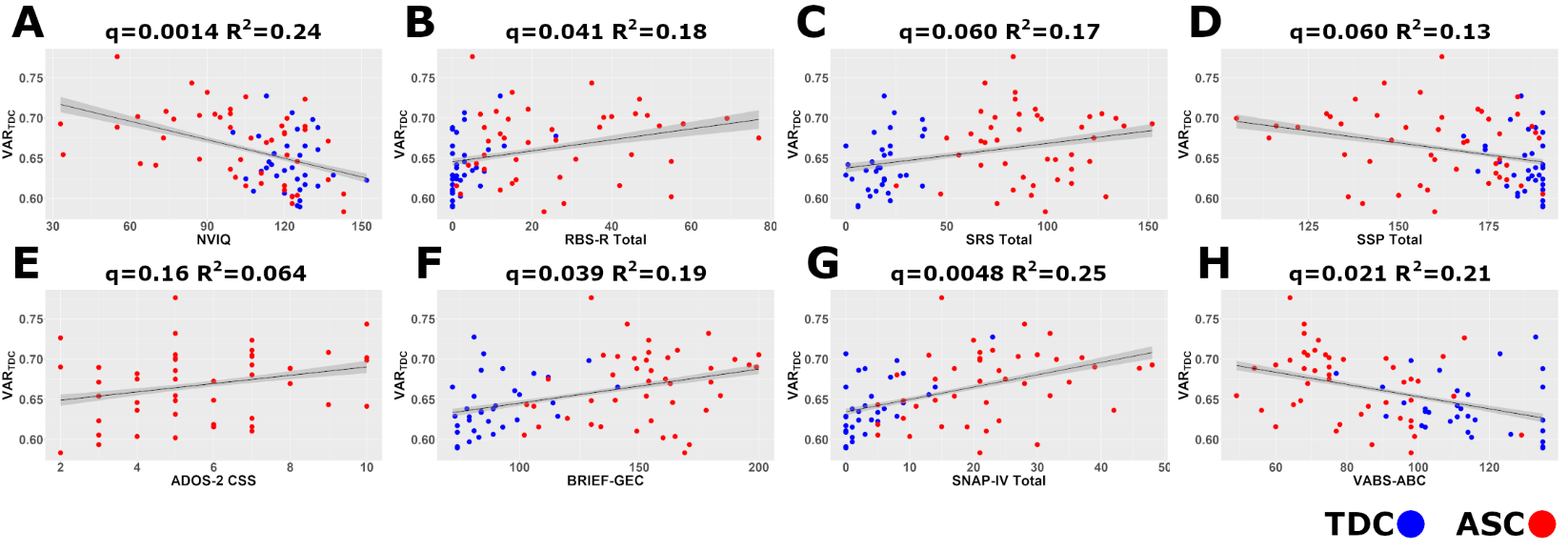
**

**Figure S7. Transdiagnostic linear correlations between TDC-reference neural idiosyncrasy (Variability_TDC_) and cognitive/behavioural measures.** Scatterplots depict relationships across all participants, controlling for age, sex, and mean FD. Mean FD was treated as a smooth term in the GAMs since its distribution was not Gaussian. Significant linear associations (FDR q<0.05) were found for NVFIQ (A), RBS-R Total (B), and SSP Total (D), BRIEF-GEC (F), SNAP-IV Total (G), and VABS-ABC (H). SRS Total (C) and ADOS-2 CSS (E) were not significantly associated. Shaded areas represent 95% confidence intervals. Notably, higher SSP scores represent fewer sensory symptoms.
